# Deep Contrastive Learning for High‐Throughput Prediction of Drug Resistance Mutations from Sequences

**DOI:** 10.1002/advs.202514899

**Published:** 2026-07-29

**Authors:** Xiaowen Hu, Pan Zhang, Shangqian Wu, Hao Sun, Minwei Li, Sophia Tsoka, Zizhang Sheng, Lei Deng

**Affiliations:** ^1^ School of Computer Science and Engineering Central South University Changsha Hunan China; ^2^ Infection Control Center Xiangya Hospital of Central South University Changsha Hunan China; ^3^ National Clinical Research Center for Geriatric Disorders Xiangya Hospital of Central South University Changsha Hunan China; ^4^ Department of Informatics King's College London London United Kingdom; ^5^ Aaron Diamond AIDS Research Center Columbia University Vagelos College of Physicians and Surgeons New York New York United States

**Keywords:** deep contrastive learning, drug resistance, drug‐target binding affinity, protein mutations

## Abstract

Mutation‐induced drug resistance challenges both pandemic surveillance and drug discovery. While experimental assays are resource‐intensive, current computational predictions remain limited by the scarcity of 3D mutant protein structures. We present DeepMutDTA, a structure‐independent model pre‐trained on 1.5 million data points to predict drug‐target affinity and uncover underlying interaction mechanisms. However, like other sequence‐based approaches, it often falls short in predicting mutant affinities due to the overwhelming sequence similarity between wild‐type (WT) and mutant (MT) targets. To bridge this gap, we introduce SimSiam‐MuTF, a novel fine‐tuning framework to enhance the detection of resistance variants by explicitly aligning latent embedding distances with the corresponding shifts in binding affinity between WT and MT targets. Compared to representative baselines, our model exhibits remarkable robustness across varied sequence identities and unseen data splits, yielding average performance gains of 2.47% (PCC) and 5.10% (SCC) in regression tasks, alongside 4.00% (AUC) and 4.17% (AUPR) in classification tasks. Applications to SARS‐CoV‐2, HIV‐1, and cancer‐related targets highlight its generalization potential and utility in informing therapeutic strategies against drug resistance. Collectively, this robust computational pipeline and fine‐tuning framework deepen our understanding of mutation‐induced resistance and may serve as a powerful platform to accelerate drug discovery against mutant targets.

## Graphical abstract

This study presents DeepMutDTA, a deep learning framework aimed at predicting mutation‐induced changes in protein‐drug interactions and prioritizing variants potentially linked to drug resistance. Trained on large‐scale data, it incorporates SimSiam‐MuTF, a label‐aware contrastive fine‐tuning strategy that encourages separation between WT and MT representations. The results provide support for drug‐resistance surveillance and therapeutic development.

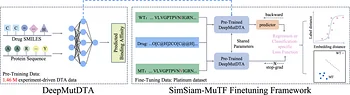

## 1 Introduction

Amino acid substitutions in proteins, particularly within the binding pocket, can substantially impact drug‐target affinity (DTA), often contributing to diminished therapeutic efficacy and the clinical emergence of drug resistance [1, 2]. Wet‐lab assays of drug‐resistant mutations are typically resource‐intensive and time‐consuming. Consequently, computational methods have emerged as scalable alternatives, facilitating the rapid evaluation of mutation‐induced changes in DTA and helping to prioritize candidates for in vitro validations [3].

Prior approaches to evaluating drug effects on mutant proteins have primarily focused on predicting changes in DTA (ΔΔG prediction). These methods, exemplified by PremPLI [4], predominantly utilize machine learning techniques to encode drug‐target complexes into latent embeddings. Other recent approaches, such as Emden [5], have reformulated ΔΔG prediction as a classification task, integrating graph neural networks and Transformer architectures to derive features from drug and protein sequences, achieving highly competitive performance. However, ΔΔG prediction primarily evaluates the relative change in binding affinity, which may constrain certain downstream applications, such as, drug repositioning for mutant proteins, where absolute affinity is often critical for therapeutic assessment.

As an alternative paradigm, DTA prediction methods have attracted considerable research attention. Sequence‐based methods generate embeddings directly from raw protein and drug sequences and subsequently integrate these representations to predict DTA [6, 7, 8, 9, 10]. Structure‐based methods leverage the three‐dimensional structural information of proteins and drugs, or their complexes, aiming to improve predictive capabilities by explicitly modeling the structural context of binding pockets [11, 12] or defining interaction interfaces to capture detailed interaction patterns [13]. Multimodal‐based methods combine heterogeneous features at multiple levels and integrate them using specialized aggregation modules to obtain unified representations, potentially enhancing binding affinity prediction [14, 15]. Given the flexibility of these frameworks, we hypothesize that utilizing DTA prediction approaches to evaluate drug effects on mutant proteins may offer enhanced adaptability and broader applicability, a direction recently explored by [16].

Intuitively, structure‐based methods appear well‐suited for understanding the impact of protein mutations on DTA, since these mutations often induce conformational changes that confer drug resistance. However, the limited availability of experimentally resolved mutant protein structures constrains the scalability of these models and can increase the risk of overfitting [17]. Although computational prediction of protein structures has significantly expanded due to advancements like AlphaFold2 [18], inherent inaccuracies in such models may compromise their reliability for predicting DTA [19, 20]. By operating independently of structural data, sequence‐based methods have emerged in recent research as a promising alternative capable of predicting drug effects on mutant proteins [16, 21]. Nevertheless, distinguishing the subtle differences between wild‐type (WT) and mutant (MT) protein sequences remains a foundational challenge, limiting the application of recent DTA models to mutant proteins. Building on recent advancements in Siamese networks for modeling subtle representational differences [22, 23, 24, 25], we hypothesize that adopting this architecture can enhance sensitivity to fine‐grained sequence variations, thereby better capturing the impact of mutations on DTA.

To address these challenges, we introduce DeepMutDTA, a sequence‐based model tailored to predict DTA within mutation‐driven drug discovery contexts. DeepMutDTA integrates FastFormer as the protein sequence encoder and MolFormer to encode drug SMILES, followed by a Top‐K attention mechanism for binding affinity prediction. Additionally, we present SimSiam‐MuTF, a mutation‐aware fine‐tuning framework designed to guide the pre‐trained DeepMutDTA in better estimating the effects of missense mutations on drug‐target interactions. Extensive evaluations indicate DeepMutDTA's robust performance and predictive capabilities following fine‐tuning. Moreover, the SimSiam‐MuTF framework shows potential adaptability, extending to additional biological contexts where minor perturbations yield significant functional impacts, including mutation‐induced effects on protein‐protein interactions (PPIs). Our evaluation supports the practical applicability and competitive performance of the fine‐tuned DeepMutDTA across diverse tasks, such as investigating resistance mechanisms, analyzing viral evolutionary trajectories, and identifying potential drug repositioning opportunities. Crucially, all evaluations were performed exclusively on datasets excluded from the pre‐training and fine‐tuning phases, reinforcing the model's generalization potential when predicting previously unseen mutations.

## 2 Results

### 2.1 Model Architecture

DeepMutDTA utilizes protein sequences and drug SMILES representations to predict binding affinity, as depicted in Figure 1A. Given the high sequence similarity between WT and MT proteins and the inherent sparsity of mutation data, DeepMutDTA implements a two‐stage training strategy comprising pre‐training and fine‐tuning phases. In the pre‐training phase, DeepMutDTA undergoes supervised learning on approximately 1.46 million experimentally derived drug‐target binding affinity data points, aiming to establish informative representations to support subsequent binding affinity predictions. Subsequently, during the fine‐tuning phase, the SimSiam‐MuTF framework is employed to refine the pre‐trained DeepMutDTA model, enhancing its capability to capture potential mutation residues and estimate the impact of amino acid substitutions on drug‐target affinity using the mutation dataset.

**FIGURE 1:**
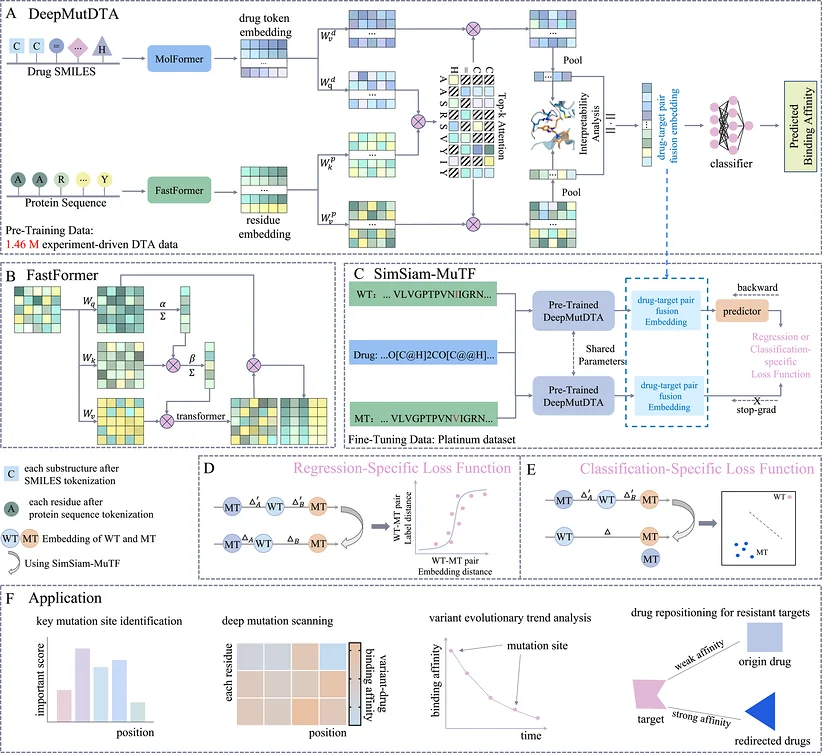
The Architecture of DeepMutDTA. (A) Schematic representation of the DeepMutDTA architecture. The model utilizes MolFormer and FastFormer to encode drug SMILES and protein sequences, respectively. A Top‐K attention mechanism is applied to model potential interactions within drug‐target pairs, followed by a two‐layer fully connected network to predict binding affinity. (B) FastFormer Architecture: This module leverages global attention to encode protein sequences, offering enhanced computational efficiency and global feature extraction compared to vanilla Transformer architectures. (C) SimSiam‐MuTF framework: During fine‐tuning, task‐specific loss functions are incorporated to refine the pre‐trained model, aiming to enhance its capacity to characterize mutation‐induced effects across regression and classification tasks. (D) Regression‐Specific Loss Function: Utilizes a rank‐based contrastive (RnC) loss designed to encourage a positive correlation between the WT–MT embedding distance and the corresponding binding affinity difference. (E) Classification‐Specific Loss Function: Employs supervised contrastive learning (SCL), aiming to maximize the embedding distance between WT and MT sequences associated with the same drug. (F) Application: Illustrates potential downstream applications of DeepMutDTA.

During the pre‐training phase, DeepMutDTA utilizes FastFormer (Figure 1B) and MolFormer [26] to encode protein sequences and drug SMILES, respectively, yielding fine‐grained embeddings for both proteins and drugs. A top‐K attention mechanism, coupled with an attention pooling strategy, is then applied to estimate importance scores for individual protein residues and drug tokens. This design aims to facilitate the model's interpretability by highlighting potential interaction sites. Finally, the resulting representations are concatenated and processed through a two‐layer fully connected network to predict binding affinity.

In the fine‐tuning phase, we introduce the SimSiam‐MuTF framework to enhance DeepMutDTA's capability to capture mutation‐specific effects (Figure 1C). SimSiam‐MuTF is adapted from the SimSiam framework [22], which was initially designed for unsupervised image feature extraction. Inspired by prior work [22, 23], we extend this framework to supervised learning scenarios by incorporating task‐specific loss functions, aiming to help the encoder recognize potential mutation sites within highly similar protein sequences. This adaptation addresses both regression and classification downstream tasks, facilitating a more robust assessment of mutation impacts. Specifically, rather than relying on conventional data augmentation methods, SimSiam‐MuTF directly leverages WT and MT sequences paired with the same drug as inputs for the online and target networks. For regression tasks, we employ Rank‐N‐Contrast (RnC) and Mean Squared Error (MSE) loss functions (Figure 1D), designed to encourage a positive correlation between the embedding distance of WT and MT proteins bound to the same drug and the corresponding differences in their binding affinities. For classification tasks, we utilize Supervised Contrastive Learning (SCL) and Binary Cross‐Entropy (BCE) loss functions (Figure 1E), aiming to maximize the embedding distance between WT and MT sequences associated with the same drug. Through these enhancements, SimSiam‐MuTF seeks to improve the predictive performance and interpretability of mutation effects on drug‐target binding affinity (detailed in Section 4).

To evaluate the broader applicability of DeepMutDTA across various scenarios, we conducted a systematic evaluation focusing on two primary dimensions: viral surveillance and targeted therapeutic discovery. We first assessed DeepMutDTA's performance under out‐of‐distribution (OOD) conditions. Subsequently, to explore its potential utility in pandemic monitoring, we applied the model to analyze the mutational landscape of the SARS‐CoV‐2 main protease and to predict potential multidrug‐resistance mutations in the HIV‐1 protease. Finally, we investigated the model's capacity to inform therapeutic strategies by performing computational drug repurposing for specific cancer‐associated mutations.

### 2.2 Performance Evaluation of DeepMutDTA on Platinum Dataset

During the pre‐training stage, we utilized a curated dataset comprising IC50 measurements extracted from BindingDB [27] and BioLip [28]. Following a systematic data filtering process (Note S2), the final dataset consisted of 1,459,913 interactions, encompassing 7,98,390 unique drugs and 16,202 protein sequences. Initial performance evaluations demonstrated that the pre‐trained DeepMutDTA outperformed competing methods. Furthermore, interpretability analyses suggested that the model's attention mechanisms could highlight the interaction regions between drugs and targets (Note S1).

Given that the Platinum dataset represents a highly comprehensive literature‐derived mutation resource, we fine‐tuned the pre‐trained DeepMutDTA model on this dataset for subsequent downstream applications. Initially, we evaluated the model's performance on the Platinum dataset by focusing on two key predictive scenarios, fine‐tuning the model independently for each: absolute binding affinity prediction and affinity change direction (ΔAffinity) prediction (detailed in Section 4.1). For binding affinity prediction, the Pearson correlation coefficient (PCC) and Spearman correlation coefficient (SCC) were employed as primary metrics, facilitating a robust assessment of both linear and monotonic relationships between predicted and experimentally observed affinities. For the ΔAffinity prediction task, the model's performance was evaluated using the area under the receiver operating characteristic curve (AUC) and the area under the precision‐recall curve (AUPR). These metrics provided valuable insights into the model's capacity to distinguish between mutation‐induced affinity alterations.

We evaluated the DeepMutDTA model and its SimSiam‐MuTF–fine‐tuned variant (DeepMutDTA‐MuTF) on the Platinum dataset using five‐fold cross‐validation (5CV). Their performance was compared against six representative baseline methods: DeepDTA [6], AttentionDTA [8], HyperAttentionDTI [9], MFE [15], TransformerCPI 2.0 [21], and DrugBAN [29]. Among these, DeepDTA, AttentionDTA, HyperAttentionDTI, and DrugBAN rely on sequence‐based architectures; MFE utilizes a multimodal approach; and TransformerCPI 2.0 is driven by a protein language model. Additionally, we expanded our comparisons to specialized methods for ΔAffinity prediction, including PremPLI [4] and Emden [5], aiming to provide a well‐rounded contextualization against existing predictive approaches. To further assess the robustness and generalization potential of DeepMutDTA, we introduced five distinct OOD evaluation scenarios designed to reflect various challenges in drug‐target binding affinity prediction. Specifically, the dataset was partitioned as follows: (1) training on single amino acid substitutions and predicting the effects of multiple substitutions; (2) split in which the training and test sets do not share any drugs; (3) split in which they do not share any proteins; (4) split in which neither drugs nor proteins are shared; and (5) split in which drug–target pairs are divided according to different protein sequence identity thresholds(10%, 20%, 30%, 40%, and 50%) (see Figure 2A–E and Note S4).

**FIGURE 2:**
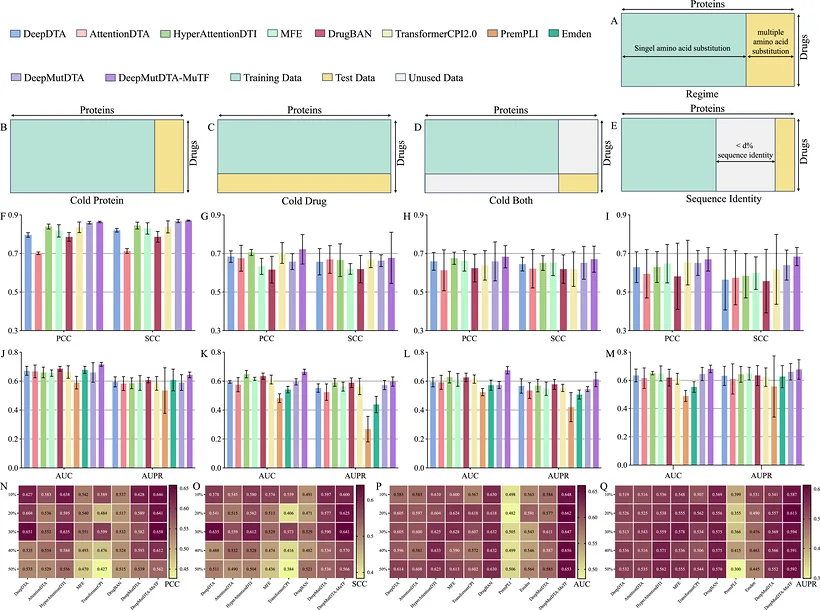
Performance comparison between DeepMutDTA and representative baseline models on the Platinum dataset. (A–E) Schematic illustration of the five train–test split strategies considered in this study: Regime, Cold Protein, Cold Drug, Cold Both, and Sequence Identity. (F–I) Performance comparison on the binding affinity prediction task across four evaluation scenarios (Regime, Cold Protein, Cold Drug, and Cold Both Scenarios). (J–M) Performance comparison on the ΔAffinity task across the same four evaluation scenarios (Regime, Cold Protein, Cold Drug, and Cold Both Scenario). (N‐Q) Performance comparison across varying sequence‐identity thresholds (10%, 20%, 30%, 40%, and 50%) of the binding affinity prediction and ΔAffinity prediction task.

Our evaluation results (Figure 2F–Q) indicate that fine‐tuning DeepMutDTA using the SimSiam‐MuTF framework yields notable performance improvements across both regression and classification tasks, with maximum gains of 8.85% in PCC, 6.44% in SCC, 20.77% in AUC, and 16.2% in AUPR. Specifically, the DeepMutDTA‐MuTF model outperformed the evaluated baseline methods across the tested conditions. Under standard five‐fold cross‐validation, DeepMutDTA‐MuTF achieved improvements of 1.36% and 0.97% in PCC and SCC, respectively, for binding affinity prediction, alongside enhancements of 0.43% (AUC) and 1.98% (AUPR) for ΔAffinity prediction (Figure S6). This positive trend extended to the challenging OOD scenarios. In the Regime Scenario, the model surpassed the next‐best methods by 2.76% (PCC) and 2.94% (SCC) on the affinity task, and by 4.16% (AUC) and 5.46% (AUPR) on the ΔAffinity task. Comparable performance gains were observed in the Cold Protein Scenario (enhancing PCC/SCC by 2.27% and 1.31%, and AUC/AUPR by 2.41% and 1.76%) and the Cold Drug Scenario (improving PCC/SCC by 1.09% and 2.60%, and AUC/AUPR by 7.16% and 5.58%). Finally, in the highly stringent Cold Both Scenario, the model maintained its advantage, recording improvements of 2.56% (PCC) and 9.67% (SCC) for affinity prediction, and 4.40% (AUC) and 4.28% (AUPR) for ΔAffinity prediction. We plot the scatter of DeepMutDTA‐MuTF as shown in Figure S7. These results suggest that DeepMutDTA‐MuTF holds a distinct advantage in OOD settings, highlighting its potential for generalization when confronted with novel, unseen samples. Among the evaluated baselines, MFE integrates protein structural, surface, and sequence information to construct a comprehensive protein representation. Despite this information‐rich feature set, its performance was less optimal in these specific tasks. We hypothesize that this may be due to the structured sparsity of the mutation data, which could yield limited or noisy supervision signals, potentially hindering the multimodal model's ability to extract reliable interaction patterns.

Moreover, assessments under conditions characterized by varying protein sequence identities (Figure 2N–Q) indicated that DeepMutDTA‐MuTF maintained relatively consistent predictive capabilities. In the binding affinity prediction task, the DeepMutDTA‐MuTF model surpassed the next‐best methods in terms of PCC by 1.23%, 5.77%, 1.06%, 4.57%, and 0.91% at sequence identity thresholds of 10%, 20%, 30%, 40%, and 50%, respectively. Corresponding improvements in SCC were 3.66%, 10.08%, 0.93%, 6.66%, and 8.09%. Similarly, for the ΔAffinity prediction task, the model yielded enhancements of 2.77%, 5.74%, 2.31%, 3.51%, and 3.52% in AUC, alongside AUPR increases of 3.06%, 8.31%, 2.69%, 2.70%, and 3.71% across the respective sequence identity levels. In this setting, we observed that neither DeepMutDTA nor the baseline methods achieved their peak performance in the high sequence‐similarity regime. We hypothesize that, although highly similar proteins often share similar functions, mutations within the same protein can exert context‐dependent, site‐specific effects on binding affinity [30]. Consequently, if a model relies primarily on global sequence homology to infer mutant protein–drug binding affinity, it may become over‐reliant on sequence cues and struggle to adequately capture context‐relevant, mutation‐induced affinity changes. By contrast, DeepMutDTA‐MuTF leverages the SimSiam framework to guide the pre‐trained model toward learning mutation‐related effects. This design encourages the model to prioritize localized mutation–interaction patterns rather than depending strictly on overall sequence similarity, thereby contributing to its favorable performance.

Collectively, the enhanced predictive performance of DeepMutDTA is likely driven by its integration of the SimSiam‐MuTF framework, a robust pre‐training strategy, and a tailored loss function. In particular, the incorporation of a supervised contrastive learning approach within the SimSiam‐MuTF framework facilitates the identification of fine‐grained feature differences induced by mutations, thereby improving the model's sensitivity to subtle yet biologically relevant changes. This architectural advantage is further supported by our ablation study (detailed in Section 2.6). Overall, our comprehensive evaluations suggest that the DeepMutDTA‐MuTF model exhibits promising robustness and generalization in evaluating binding affinity changes due to mutations across various evaluated scenarios. Furthermore, its capacity to process unseen proteins, drugs, and challenging sequence similarity contexts without requiring additional structural input highlights DeepMutDTA's potential as a valuable computational resource for drug resistance prediction.

### 2.3 Identification of Drug‐Resistant Variants of the SARS‐CoV‐2 Main Protease Throughout the COVID‐19 Pandemic Using DeepMutDTA‐MuTF

The management of COVID‐19 continues to present challenges due to the emergence of potentially drug‐resistant SARS‐CoV‐2 variants. Nirmatrelvir, an antiviral agent specifically targeting the main protease ($M^{\text{pro}}$) of SARS‐CoV‐2, has been widely deployed for COVID‐19 treatment. However, the inherent mutation rate of the virus can lead to structural alterations within the $M^{\text{pro}}$ substrate‐binding pocket, which may confer resistance to nirmatrelvir [30, 31]. To explore the practical utility of our model beyond theoretical evaluation, we applied DeepMutDTA‐MuTF to retrospectively analyze the impact of documented $M^{\text{pro}}$ mutations on drug binding affinity over the course of the pandemic.

We initially evaluated the performance of the DeepMutDTA‐MuTF model using the SARS‐CoV‐2 dataset. The SARS‐CoV‐2 dataset [31] contains experimentally determined binding affinity data between the SARS‐CoV‐2 $M^{\text{pro}}$ and three structurally distinct antiviral drugs: GC‐376, PF‐00835231, and PF‐07321332 (detailed in Section 4.2). The model, trained and validated on the Platinum dataset, was assessed on both absolute binding affinity and ΔAffinity prediction tasks. Additionally, we compared its predictive performance against molecular docking results derived from AutoDock Vina. As illustrated in Figure 3A, B, DeepMutDTA‐MuTF outperformed the evaluated baseline methods on this test set. Specifically, it yielded notable improvements over the next‐best method, increasing PCC and SCC by 17.53% and 13.14%, respectively, for binding affinity prediction, and enhancing AUC and AUPR by 12.58% and 6.81% for ΔAffinity prediction. One‐way ANOVA confirmed that these improvements in PCC and AUC were statistically significant. Furthermore, the SimSiam‐MuTF regression objective was designed to encourage a positive correlation between the Euclidean distance of the generated embeddings and the corresponding label differences. To assess this, we computed the embedding distances for DeepMutDTA‐MuTF alongside six baseline methods, subsequently calculating their respective PCC and SCC against the true labels. The results (Figure 3C) indicate that our model substantially surpassed the next‐best method, achieving relative gains of 54.77% and 52.42% in PCC and SCC. These findings suggest that the framework successfully aligns the feature space with the functional impacts of mutations, highlighting its potential to extract robust, biologically relevant representations.

**FIGURE 3:**
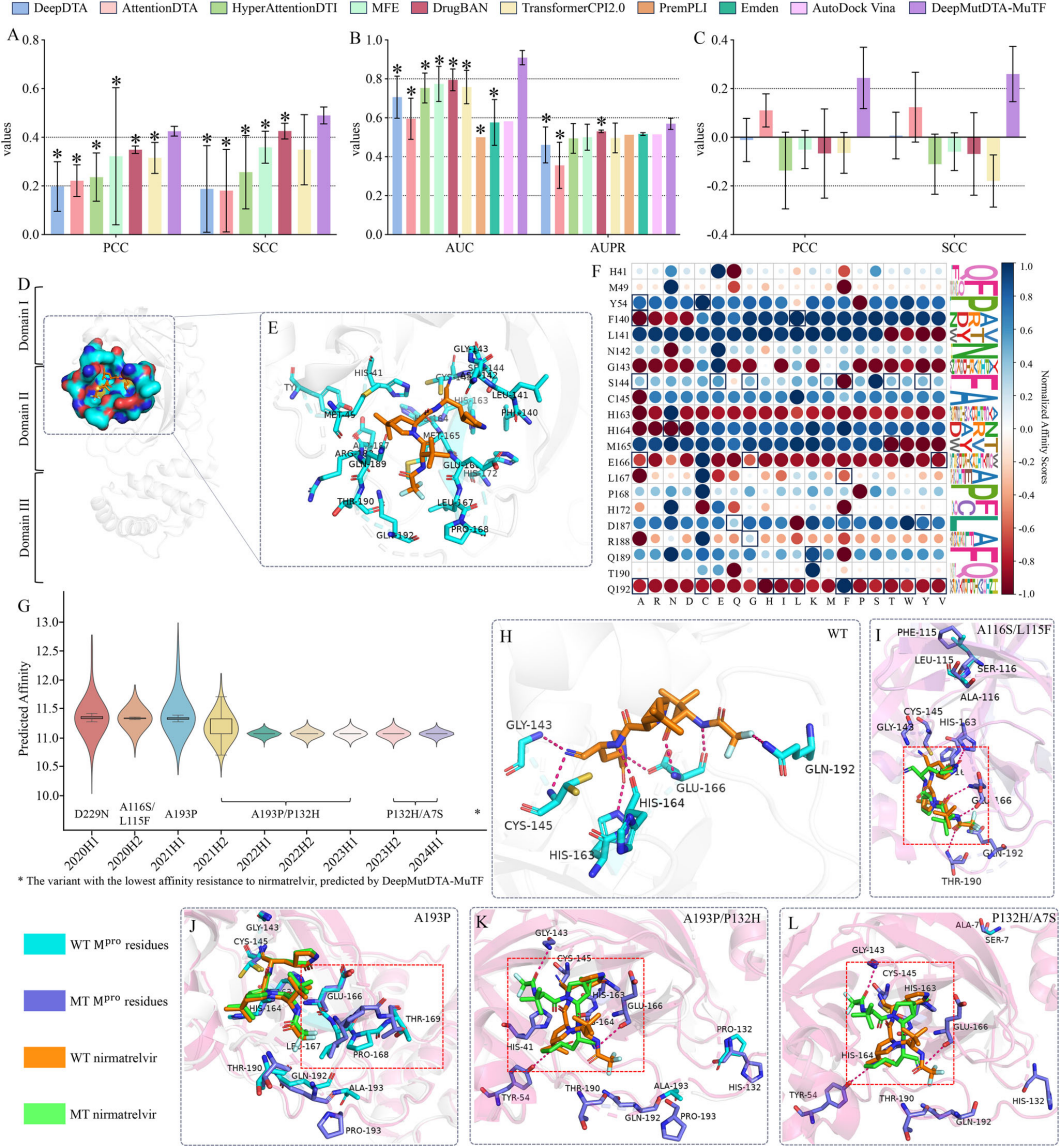
Evaluating the mutational landscape of SARS‐CoV‐2 $M^{\text{pro}}$ using DeepMutDTA‐MuTF. (A,B) performance evaluation of DeepMutDTA‐MuTF with other baseline methods on the SARS‐CoV‐2 independent test set in the binding affinity prediction task and ΔAffinity prediction task. Statistical significance is indicated as follows: *p < 0.05. (C) correlation evaluation between the embedding generated by the models and the distance of labels. (D) The binding pocket of the SARS‐CoV‐2 $M^{\text{pro}}$ bound to the drug nirmatrelvir (PDB ID: 7VH8 [32]). (E) Key binding sites within the SARS‐CoV‐2 $M^{\text{pro}}$ that interact with nirmatrelvir. (F) Heatmap and sequence logo depicting the predicted binding affinity of each mutant strain with nirmatrelvir, generated using the DeepMutDTA‐MuTF model by substituting amino acids at identified mutation sites in the $M^{\text{pro}}$ with the 20 standard amino acids. Lower scores indicate weaker binding affinity, with larger amino acid letters representing higher significance. The black markers indicate the verified resistance mutations in the Stanford Coronavirus Antiviral & Resistance Database. (G) Violin plot showing predicted binding affinity between SARS‐CoV‐2 $M^{\text{pro}}$ variants and nirmatrelvir across the GISAID database. (H‐L) Structural representations of the WT (H), A116S/L115F (I), A193P (J), A193P/P132H (K), and P132H/A7S (L) forms of $M^{\text{pro}}$ in complex with nirmatrelvir.

Next, we applied the DeepMutDTA‐MuTF model to predict potential mutation residues in the SARS‐CoV‐2 $M^{\text{pro}}$ that may contribute to nirmatrelvir resistance. Our analysis began with the identification of the drug‐binding pocket within the $M^{\text{pro}}$‐nirmatrelvir complex (PDB ID: 7VH8 [32]) (Figure 3D). Guided by existing literature [32], we selected 21 critical binding sites within the pocket (Figure 3E) and systematically substituted each position with the 20 standard amino acids. Using DeepMutDTA‐MuTF, we predicted the binding affinity of each SARS‐CoV‐2 variant to nirmatrelvir, normalized the predictions, and visualized the results using a heatmap and sequence logo (Figure 3F). In this context, lower predicted binding affinity scores are indicated to reflect a higher likelihood of drug resistance, with candidates for resistant mutations prominently displayed in the sequence logo. To evaluate these predictions, we compared our results against experimentally documented nirmatrelvir‐resistant mutations in $M^{\text{pro}}$ from the Stanford Coronavirus Antiviral & Resistance Database [33]. We set 0.0 as the threshold for the normalized affinity scores, where values below zero were considered indicative of a potential resistance residue. As shown in Figure 3F, we computed the confusion matrix for each site (Table S2) and aggregated the counts. The overall confusion matrix yielded TP = 18, FP = 157, TN = 230, and FN = 15, corresponding to an Accuracy of 0.591, a Recall of 0.546, and a Specificity of 0.594. These findings suggest that the DeepMutDTA‐MuTF model can provide valuable computational screening for drug‐resistant mutations, underscoring its potential utility in supporting drug resistance surveillance and therapeutic design.

We subsequently investigated the temporal accumulation of mutations in the $M^{\text{pro}}$ enzyme to better understand the virus's evolutionary dynamics. Specifically, we applied the DeepMutDTA‐MuTF model to sequence data from GISAID (Global Initiative on Sharing All Influenza Data) [34] to assess its utility in monitoring mutational trends. We retrieved $M^{\text{pro}}$ sequences spanning January 2020 to February 2024 and applied the following filtering criteria: (1) exclusion of incomplete sequences (shorter than 29,000 nucleotides), (2) removal of low‐coverage sequences (>5% undefined bases), and (3) omission of entries lacking collection dates. This curation process yielded a final dataset of 14,261,299 sequences. Using DeepMutDTA‐MuTF, we estimated the binding affinities between these SARS‐CoV‐2 variants and nirmatrelvir at six‐month intervals, visualizing the distributions with violin plots (Figure 3G). Over the observed time‐frame, the average predicted binding affinity exhibited a gradual decline, a trend that may reflect the progressive accumulation of resistance‐associated mutations. For each interval, we isolated the variants exhibiting the lowest predicted binding affinities, classifying them as candidate resistant mutants for further trend analysis (Table S3). Among these candidates, the A193P substitution aligns with a previously documented, experimentally validated resistance mutation [30]. For variants lacking resolved experimental structures, we introduced the corresponding amino acid substitutions in silico using PyMOL, generated complex structures via molecular docking with AutoDock Vina, and characterized target‐drug interactions using PLIP [35].

In the first year, dominant mutations included D229N and A116S/L115F (Figure 3I). Compared to the WT structure (Figure 3H), the A116S/L115F mutation altered the conformation of nirmatrelvir within the binding pocket, disrupting key hydrogen bonds at GLY‐143, CYS‐145, HIS‐164, and GLU‐166, which contributed to resistance. As mutation trends evolved, A193P became the primary mutation in early 2021. Structural analysis by [30] indicated that the A193P mutation‐induced conformational changes in residues P167–T169, impacting hydrogen bond formation and destabilizing the protein, leading to drug resistance (Figure 3J). Over the following two years, additional mutations gained prominence. The combined A193P/P132H mutation (Figure 3K) exhibited further conformational changes in nirmatrelvir and additional hydrogen bond disruptions within the binding pocket compared to A193P alone, retaining bonds only at GLY‐143 and GLU‐166. Two new bonds formed at TYR‐54 and HIS‐41, causing nirmatrelvir to progressively shift out of the $M^{\text{pro}}$ binding pocket. Eventually, the P132H/A7S mutation (Figure 3L) became dominant. Compared to the P132H mutation alone [36], this combination induced significant conformational alterations in nirmatrelvir, almost leading to its dissociation from the binding pocket. The disruption of the hydrogen bond at HIS‐41 conferred even greater resistance.

### 2.4 Predicting Potential HIV‐1 Resistance Mutation Using DeepMutDTA‐MuTF

Recent advancements in antiretroviral therapy have largely transitioned HIV‐1 infection into a manageable chronic condition. However, the virus's high mutation rate continues to drive the emergence of drug resistance, potentially compromising therapeutic efficacy. While combination therapy, typically involving three or more antiretroviral agents, offers a more robust defense than monotherapy, resistance mutations can still accumulate, occasionally giving rise to multidrug‐resistant HIV‐1 strains. Consequently, characterizing the impacts of amino acid substitutions and investigating potential mutation variants that confer multidrug resistance remain highly valuable for informing therapeutic strategies and improving patient outcomes. To contribute to this ongoing effort, we applied the DeepMutDTA‐MuTF model in conjunction with interpretability techniques, aiming to extract computationally derived insights into HIV‐1 resistance mechanisms triggered by amino acid substitutions.

Initially, we evaluated the performance of the DeepMutDTA‐MuTF model using an independent HIV dataset. Sourced from the Stanford HIV Database, this dataset includes 95 literature‐documented protease inhibitor resistance records, characterizing interactions between the HIV‐1 protease and eight antiviral drugs. To investigate the cross‐domain generalization potential of DeepMutDTA‐MuTF, the model was trained and validated on the Cancer dataset (detailed in Section 4.3) before being tested on the HIV dataset (detailed in Section 4.4). Performance was quantitatively assessed using AUC and AUPR metrics. As illustrated in Figure 4A, DeepMutDTA‐MuTF achieved higher scores than the evaluated baseline models, surpassing the next‐best methods by 12.87% in AUC and 17.10% in AUPR. One‐way ANOVA indicates that these performance differences are statistically significant relative to most compared methods. These results suggest a promising ability to generalize across domains, even when the model is trained on a distinct disease context such as cancer, highlighting the framework's potential for adaptability across diverse therapeutic areas.

**FIGURE 4:**
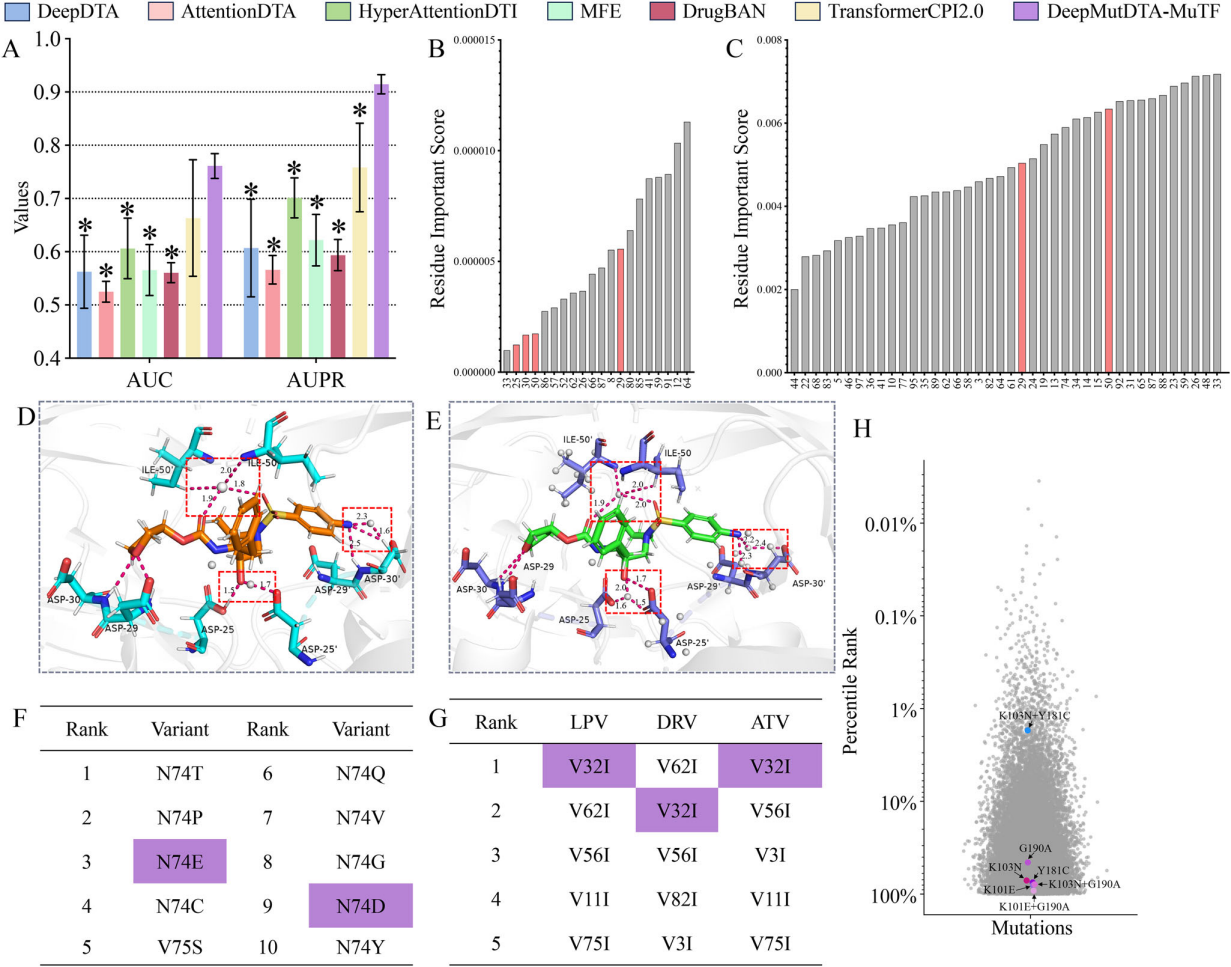
Evaluation of potential HIV‐1 drug‐resistance mutations predicted by DeepMutDTA‐MuTF. (A) Comparative predictive performance on the independent HIV dataset (*p < 0.05). (B, C) Reverse bar charts displaying the predicted contribution of specific residues to resistance, based on importance scores derived from DeepMutDTA‐MuTF (B) and TransformerCPI2.0 (C). Clinically documented resistance‐associated sites are highlighted in red. (D, E) Structural modeling of interactions between APV and key protease residues across wild‐type and mutant states. (F) Top‐ranked candidate HIV‐1 capsid mutations predicted to confer lenacapavir (LEN) resistance, identified via in silico deep mutational scanning. (G) Highest‐scoring protease mutations predicted to impair binding affinity across three distinct antiviral drugs. Validated resistance variants are denoted in purple. (H) Predicted resistance percentile ranks for isolated and combined HIV‐1 mutations.

Subsequently, we investigated the resistance mechanism by comparing the HIV‐1 protease (PDB ID: 4JEC [37]) and its mutant variant (PDB ID: 5T8H [38]) in complex with the antiviral drug amprenavir (APV). These structural conformations represent interactions under distinct conditions, with 5T8H characterizing potential resistance mechanisms induced by the triple mutation (V32I, I47V, V82I). By utilizing the WT and MT protease sequences along with drug SMILES as inputs, we derived importance scores (detailed in Section 4.8) for each residue to evaluate their contribution to the binding affinity. In this context, higher scores were interpreted as indicative of sensitivity‐related sites, while lower scores suggested a greater likelihood of involvement in resistance. Figure 4B, C and Figure S8 illustrate the ranked per‐residue importance scores for the WT and MT protein, respectively. The analysis revealed importance score shifts for the three mutated residues, transitioning from high sensitivity in the WT to high resistance in the MT. For the HIV‐1 protease bound to APV, the residues with the highest importance scores included Leu90, Leu38, Ala22, Asp25, and Leu5. Notably, DeepMutDTA‐MuTF identified Asp25 as a primary sensitivity site (ranked 3rd) (Figure S8A). This finding is consistent with prior structural evidence showing that the strongest hydrogen bonds are formed between the central hydroxyl group of APV and the carboxylic groups of Asp25 and Asp25' [37]. In comparison, while TransformerCPI2.0, the strongest baseline in this setting, also recognized Asp25 as a sensitivity site, it ranked Asp25 significantly lower at the 19th position (Figure S8B). For the triple mutation (V32I, I47V, V82I), DeepMutDTA‐MuTF highlights key residues such as Asp25 (ranked 2nd in resistance importance), Asp30 (3rd), Ile50 (4th), and Asp29 (13th) (Figure 4B). As visualized in the 3D structural models (Figure 4D, E), this predicted resistance aligns with weakened hydrogen‐bonding interactions, which are potentially driven by increased spatial distances involving these critical residues in the mutant state. For comparison, we evaluated the residue importance scores generated by TransformerCPI2.0 (Figure 4C), which ranked Asp29 and Ile50 lower, at the 22nd and 30th positions, respectively. This indicates that DeepMutDTA‐MuTF not only achieves the best overall performance but also shows a better capability in capturing critical residues, providing insights into the resistance mechanisms driven by multiple mutations.

These findings suggest that DeepMutDTA‐MuTF can capture critical residues associated with mutation‐driven resistance. To further examine the potential influence of homologous HIV‐1 protease sequences in the pre‐training data on the observed residue‐importance patterns, we performed an additional overlap‐removal analysis. Specifically, we removed 5594 HIV‐1 protease entries from the pre‐training dataset and retrained the model using the resulting refined dataset, which contained 1,454,319 non‐HIV‐1 protein data points corresponding to 16,168 unique proteins. We then used Clustal Omega to calculate the sequence identity between HIV‐1 protease and each of the remaining proteins in the refined dataset. The distribution of sequence identity ranges is shown in Figure S9A. Among the 16,168 proteins, 48 proteins showed sequence identity in the range of 15% –20%, 631 proteins in the range of 10% –15%, 1,116 proteins in the range of 5% –10%, and 14,373 proteins in the range of 0% –5%. We further visualized the top five most similar protein sequences in Figure S9C–G and compared them with HIV‐1 protease shown in Figure S9B. Together, these results suggest that the refined dataset contained no proteins with substantial sequence similarity to HIV‐1 protease, thereby reducing the possibility that the observed residue‐importance patterns were driven by direct sequence overlap in the pre‐training data. Using the retrained model, we re‐calculated the residue importance scores for HIV‐1 protease in complex with APV (PDB ID: 5T8H [38]). As shown in Figure S10, mechanistically critical active‐site and flap residues remained highly prioritized by the retrained model, ranking 1st for Asp29, 2nd for Asp30, 3rd for Asp25, and 25th for Ile50. These residue‐importance patterns are broadly consistent with those obtained from the original DeepMutDTA‐MuTF model, which ranked the same residues 13th, 3rd, 2nd, and 4th, respectively (Figure 4B). These results suggest that the model's identification of critical resistance‐related residues is not primarily driven by memorization of homologous HIV‐1 protease sequences, but rather reflects generalizable structural and sequence patterns learned during training.

Furthermore, we leveraged DeepMutDTA‐MuTF to perform in silico deep mutational scanning, aiming to identify variants conferring potential resistance across multiple antiviral drugs. Specifically, we focused on three critical HIV‐1 targets and their respective inhibitors: lenacapavir (LEN) against the capsid protein; lopinavir (LPV), darunavir (DRV), and atazanavir (ATV) against the protease, and doravirine (DOR) against the reverse transcriptase (RT). For LEN, we used DeepMutDTA‐MuTF to estimate the binding affinities for all potential single‐residue substitutions of HIV‐1 capsid and ranked the variants by their predicted affinities. As illustrated in Figure 4F (detailed predictions are provided in Table S4), DeepMutDTA‐MuTF highlighted mutations at N74 in the top 10 resistant mutations. This aligns with recent findings [39] demonstrating N74 as a critical site during the evolutionary pathways driving in vitro HIV‐1 LEN resistance. Specifically, N74E (ranked 3rd) [39] and N74D (ranked 9th) [40] have been validated as resistance mutations. For LPV, DRV, and ATV, we ranked all single residue mutations in HIV‐1 protease according to their predicted affinities to the three drugs. We identified the top five candidates for each drug (Figure 4G). Notably, the V32I variant emerged as a top‐ranked candidate across all three drugs, a prediction highly consistent with established clinical evidence [41, 42, 43, 44, 45]. These findings strongly support the utility of DeepMutDTA‐MuTF in computationally screening for resistance‐associated mutations.

While single‐residue substitutions provide foundational insights, clinical drug resistance frequently emerges through the complex interplay of multiple concurrent mutations. Epistasis is widely recognized as a crucial factor in driving this higher‐order resistance. But currently experimentally characterized mutations with epistasis are rare for APV, LEN, LPV, DRV, and ATV. To explore the capability of DeepMutDTA‐MuTF in predicting epistatic interactions, we shifted our focus to the third target, HIV‐1 reverse transcriptease (RT), and its associated non‐nucleoside reverse transcriptase inhibitor (NNRTI) doravirine (DOR), which have epistasis mutations characterized [46]. We examined key residues situated within the primary drug‐binding pocket: L100, K101, K103, V106, V179, Y181, Y188, G190, F227, W229, L234, P236, and Y318 [47]. To investigate potential epistatic effects, we systematically substituted each site with the 19 standard amino acids and subsequently generated all possible pairwise combinations. This comprehensive mutagenesis approach yielded a total of 28,405 single and double variants, for which DeepMutDTA‐MuTF was utilized to predict the corresponding resistance probabilities. To evaluate relative drug susceptibility, these probabilities were converted into percentile ranks, where a lower percentile indicates higher predicted resistance (Figure 4H). Specifically, DeepMutDTA‐MuTF assigned K103N+Y181C a markedly high resistance association, with a percentile rank to the extreme top 1.72% of all predictions. In contrast, its constituent single mutations, K103N and Y181C, were ranked only 71.40% and 74.88%, respectively. This pronounced upward shift in ranking aligns closely with existing literature, in which the K103N, Y181C, and K103N+Y181C variants were generated in an HIV‐1 NL4‐3 backbone by site‐directed mutagenesis and evaluated using a TZM‐bl cell‐based phenotypic susceptibility assay [46]. These experiments showed that while the isolated single mutations conferred only marginal resistance to DOR (fold changes of 1.4 and 1.8, respectively), their co‐occurrence acted synergistically to substantially increase resistance (FC = 4.9). These results suggest that the model is capable of capturing such non‐linear epistatic effect. To facilitate further investigation, the top 20 predicted combinatorial variants exhibiting potential epistatic effects are detailed in Table S5. Conversely, when evaluating other combinatorial variants such as the double‐mutants K103N+G190A and K101E+G190A, the model generally maintained their low resistance rankings, placing them at 78.63% and 92.00%, respectively. This is consistent with clinical evidence indicating that these specific multi‐point mutations remain sensitive to DOR [48]. The distribution of the detailed predicted resistance probabilities is provided in Figure S11. Although the absolute predicted resistance probabilities differed modestly among these variants, DeepMutDTA‐MuTF showed a stronger ability to rank their relative resistance potentials across the broader mutational space. Compared with the exact predicted probability values, this ranking‐based interpretation may provide more practical guidance for downstream biological experiments by helping prioritize candidate variants for further validation. This is also consistent with other analyses in the manuscript, showing that mutation ranking is informative for resistant mutation identification. Collectively, these findings suggest that DeepMutDTA‐MuTF may capture aspects of the complex conformational and functional contexts associated with synergistic drug resistance, and could serve as a potential tool for prioritizing resistance‐associated mutations for downstream biological investigation.

### 2.5 Predicting Drug Responses Against Cancer‐Associated Mutant Proteins via DeepMutDTA‐MuTF

In previous sections, we demonstrated that the DeepMutDTA‐MuTF can highlight potential mutation residues and achieve favorable predictive performance in viral protein mutation contexts. In the clinical oncology setting, drug resistance arising from high mutation rates in tumors frequently compromises patient prognosis. Consequently, the computational screening and evaluation of therapeutic agents targeting resistance‐associated mutations remain highly valuable for advancing drug development and improving clinical outcomes. To assess the broader applicability and translational potential of DeepMutDTA‐MuTF, we extended its application to the analysis of tumor‐specific protein mutations.

We first assessed the generalization potential of DeepMutDTA‐MuTF on an independent test set derived from the curated cancer dataset (detailed in Section 4.3). As illustrated in Figure 5A–C and Figure S12, DeepMutDTA‐MuTF consistently outperformed the strongest baseline methods across all evaluated datasets. Specifically, it yielded improvements in AUC and AUPR of 15.77% and 16.16% on the FGFR2 dataset, 22.46% and 24.15% on AR, and 28.37% and 32.33% on ALK. This positive trend extended to the remaining datasets, with the model recording AUC and AUPR enhancements of 30.01% and 34.55% on ABL1, 17.86% and 28.05% on EGFR, 20.22% and 34.71% on Merge_10, and 13.42% and 33.5% on Merge_20. One‐way ANOVA confirmed that these performance improvements were statistically significant relative to all evaluated baseline methods across the tested metrics. These results highlight DeepMutDTA‐MuTF's stable performance and its enhanced capacity to capture binding affinity variations compared to prior models. In classification tasks, the SimSiam‐MuTF approach is designed to maximize the distance between the embeddings of WT and MT complexes for the same drug generated by DeepMutDTA‐MuTF. To examine this feature space representation, t‐SNE visualizations of the embeddings were generated for drug–protein interactions involving FGFR2 (Figure 5D), AR (Figure 5E), ALK (Figure 5F), ABL1 (Figure S13A), and EGFR (Figure S13B), with each subset containing over 10 interactions. The visualization results indicated an observable separation between WT‐drug and MT‐drug embeddings, with the MT‐drug representations tending to form distinct clusters. This clustering pattern suggests that DeepMutDTA‐MuTF is capable of capturing subtle feature differences between WT and MT proteins while simultaneously grouping shared characteristics among the MT variants.

**FIGURE 5:**
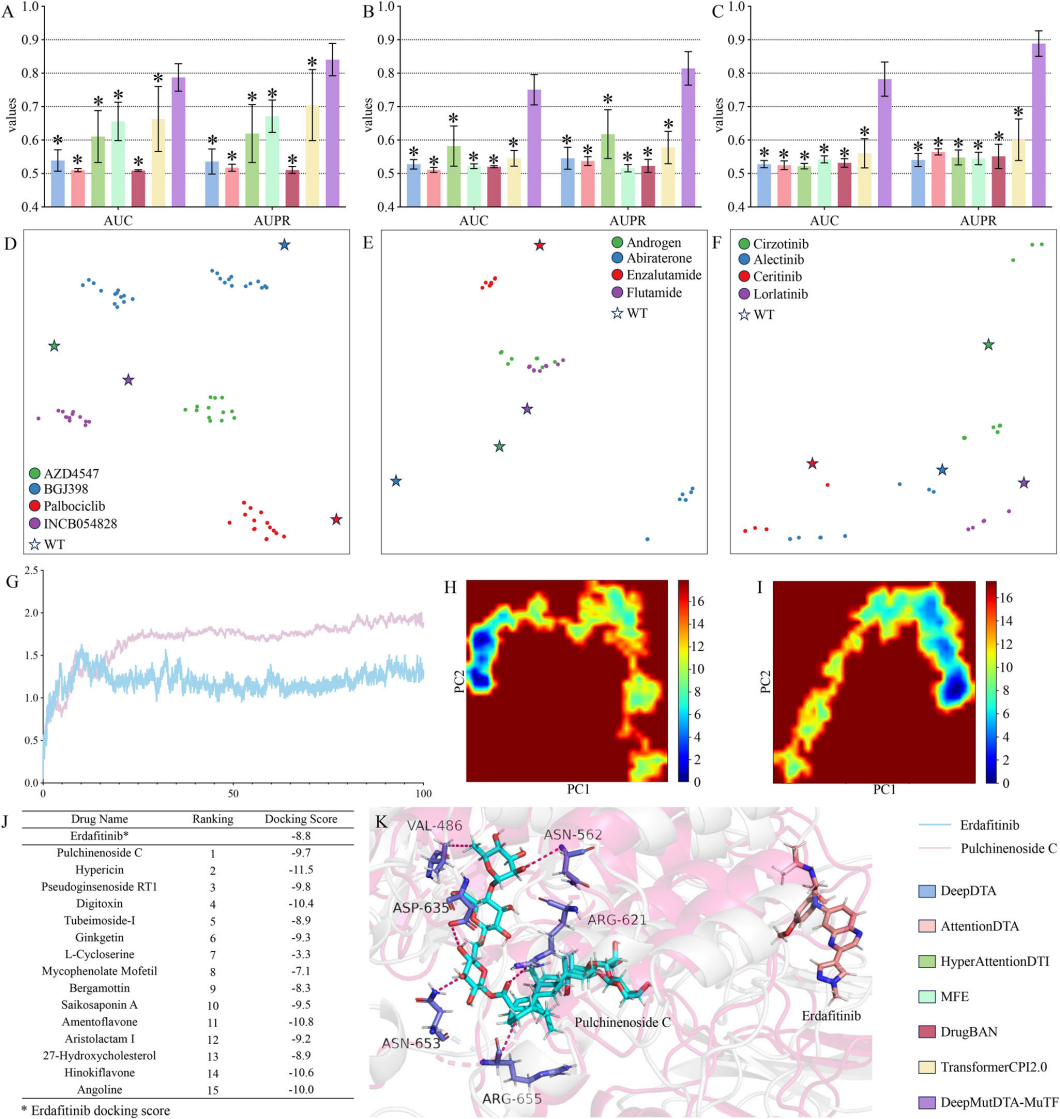
Computational evaluation of potential drug responses against cancer‐associated mutant proteins using DeepMutDTA‐MuTF. (A–C) Performance evaluation of the model on the FGFR2 (A), AR (B), and ALK (C) datasets. Statistical significance is denoted as *p < 0.05. (D–F) t‐SNE embedding visualizations for the FGFR2 (D), AR (E), and ALK (F) datasets, illustrating the learned feature separation between WT and MT protein–drug interactions. (G‐I) Molecular dynamics (MD) simulation analyses, presenting root‐mean‐square deviation (RMSD) trajectories (G) and free energy landscape profiles for Erdafitinib (H) and Pulchinenoside C (I). (J) Predicted molecular docking scores of candidate compounds against the FGFR3 S249C mutant. (K) Modeled 3D structural representations comparing the interactions of FGFR3 S249C bound to Erdafitinib and the top‐ranked drug candidate, as evaluated over 100‐ns MD simulations.

We further investigated the potential utility of DeepMutDTA‐MuTF in drug repositioning for resistance‐associated cancer protein mutations. FGFR3 mutations and fusions are prevalent genomic alterations in urothelial carcinoma that often correlate with clinical responsiveness to the FDA‐approved kinase inhibitor erdafitinib. A clinical study by [49] involving 1,421 urothelial carcinoma cases reported FGFR2/3 alterations as predictive markers for erdafitinib response in 27.5% (414 of 1,507 tumors), with FGFR3 S249C identified as the most common alteration. To explore potential therapeutic options for prevalent mutations such as FGFR3 S249C, we conducted a virtual screening campaign against a natural compound library (MedChemExpress). In the absence of an experimentally determined structure, the 3D conformation of FGFR3 S249C was modeled using ESMFold [50], followed by molecular docking via AutoDock Vina 2.0 [51]. The top 15 drug candidates prioritized by DeepMutDTA‐MuTF are shown in Figure 5J, using erdafitinib's docking score (‐8.8) as a reference benchmark. Among these candidates, 12 exhibited more favorable docking scores than erdafitinib, suggesting the model's capacity to identify potentially viable alternative compounds. To further evaluate the top‐ranked candidate, Pulchinenoside C, we performed molecular dynamics (MD) simulations to compare its theoretical binding stability with that of erdafitinib (detailed in Section 4.14). As shown in Figure 5G–I, over the course of the 100‐ns MD simulations, RMSD analysis indicated that the simulated system reached a relative equilibrium after approximately 20 ns. The average short‐range Coulombic interaction energy (Coul‐SR) for Pulchinenoside C was calculated at ‐81.0554, compared to ‐20.6087 for erdafitinib, suggesting that Pulchinenoside C forms stronger electrostatic interactions within the modeled pocket. Free energy landscape (FEL) analysis via principal component analysis (PCA) indicated that Pulchinenoside C populated a deeper and more confined global minimum basin than erdafitinib. This profile implies a more stable conformational ensemble in silico, which could translate to more favorable binding to this specific mutation. Representative conformations corresponding to the global minimum on the FEL were extracted from the trajectory for further inspection. We utilized PLIP [52] to characterize the interactions within these conformations (Figure 5K). In these models, Pulchinenoside C appeared to establish a more extensive non‐covalent interaction network, forming contacts with Val‐486, Asn‐562, Arg‐621, Asp‐635, and Asn‐653. By contrast, erdafitinib did not exhibit corresponding interactions at these specific residues in our simulations.

However, we must acknowledge an important limitation in that wet‐lab experiments remain the gold standard and are indispensable for verifying drug repurposing candidates. While 100‐ns MD simulations are widely employed for preliminary validation, they cannot fully replace biological assays. Instead, our pipeline is designed to serve as a complementary approach. DeepMutDTA‐MuTF acts as a cost‐effective preliminary filter to narrow down potential drug candidates, followed by MD simulations to further increase prediction confidence. Ultimately, this computational framework aims to provide targeted guidance for wet‐lab studies, thereby reducing experimental blindness and minimizing trial‐and‐error costs. Collectively, the results suggest that the framework can serve as a valuable computational tool for predictive virtual drug screening, offering promising avenues for future exploration in precision oncology.

### 2.6 Ablation Studies Identify Critical Components Enhancing DeepMutDTA Performance

In the preceding sections, we observed that DeepMutDTA exhibited favorable predictive performance across the Platinum, SARS‐CoV‐2, HIV, and Cancer datasets. To evaluate the contributions of individual components to the model's overall capabilities, we conducted an ablation study. Specifically, we evaluated four distinct modules within the DeepMutDTA architecture: the task‐based loss function (MSE for regression and BCE for classification), the task‐specific loss function (RnC for regression and SCL for classification), the SimSiam‐MuTF framework, and the pre‐training module.

Using the Platinum dataset for regression and the FGFR2 dataset for classification (detailed results in Table S6), we analyzed the impact of these components. Our findings suggest that relying on a single loss function generally yields sub‐optimal results across both task types. Combining task‐based and task‐specific loss functions yielded observable improvements in predictive stability compared to employing the task‐specific loss alone. This implies that while the task‐based loss helps align predictions with observed labels, the task‐specific loss further refines the embeddings to capture task‐relevant features. Furthermore, integrating the SimSiam‐MuTF framework contributed to additional performance gains, achieving increases of 7.66% in PCC and 6.61% in SCC for the regression task, alongside enhancements of 2.26% in AUC and 2.00% in AUPR for the classification task. These outcomes highlight the potential value of the SimSiam‐MuTF framework in optimizing feature representations. Additionally, the pre‐training module proved highly beneficial, particularly for classification, yielding notable improvements of 5.81% in AUC and 8.64% in AUPR.

SimSiam‐MuTF can serve as a plug‐and‐play tool, facilitating potential integration with existing models across different tasks. To explore its broader applicability, we evaluated the performance changes when applying the SimSiam‐MuTF strategy to the baseline methods (DeepDTA, AttentionDTA, HyperAttentionDTI, MFE, DrugBAN, and TransformerCPI) using the Platinum and FGFR2 datasets for regression and classification tasks, respectively. The results (Table S7) indicate that incorporating SimSiam‐MuTF consistently enhances baseline performance across all tested architectures. In the regression setting, we observed steady improvements, with maximum relative gains of 3.72% in PCC and 3.14% in SCC (achieved by HyperAttentionDTI). The performance boosts were notably more pronounced in the classification setting, particularly for attention‐based models. For instance, the integration of SimSiam‐MuTF yielded substantial enhancements for AttentionDTA (increasing AUC by 14.9% and AUPR by 11.42%) and HyperAttentionDTI (improving AUC by 10.49% and AUPR by 13.41%). These empirical findings suggest that the SimSiam‐MuTF framework offers a versatile, architecture‐agnostic approach capable of optimizing feature representations and boosting the predictive performance of various existing models.

We subsequently extended our evaluation to protein–protein interaction (PPI) prediction, curating two datasets (PPI_1102 and PPI_1402; detailed in Section 4.5). To encode the protein sequences, we tested four representative sequence‐based encoders: CNN, Transformer, DNN, and RCNN (Figure S14B–E). The resulting sequence embeddings for interacting pairs were concatenated to form fused representations. A two‐layer fully connected network was then utilized to predict binding affinity (Figure S14A). Each baseline encoder was fine‐tuned within the SimSiam‐MuTF framework, effectively substituting the pre‐trained DeepMutDTA module (Figure 1C). We compared model performance using a standard regression loss function (MSE) versus the SimSiam‐MuTF framework. As detailed in Table S8, integrating the SimSiam‐MuTF framework led to consistent performance gains across all evaluation metrics for the four tested architectures. The Transformer‐based method exhibited the most notable enhancements, improving PCC and SCC by 3.06% and 4.06% on the PPI_1102 dataset, and by 3.17% and 4.61% on the PPI_1402 dataset, respectively. These findings suggest that the SimSiam‐MuTF framework offers a versatile approach for optimizing feature extraction models, potentially facilitating enhanced performance in diverse predictive tasks.

## 3 Discussion

High‐throughput and accurate assessment of pathogen mutations that confer drug resistance remains a challenging yet essential task for advancing therapeutic development and strengthening pandemic surveillance. As pathogens continuously evolve under selective pressure, resistance‐associated substitutions can rapidly compromise drug efficacy, creating an urgent need for computational models capable of adapting to newly emerging sequences with minimal delay. To address this challenge, we propose a sequence‐based drug–target binding affinity (DTA) prediction framework, DeepMutDTA, coupled with a mutation‐oriented, label‐aware contrastive learning strategy, SimSiam‐MuTF. This framework is designed to guide a pre‐trained DeepMutDTA model toward capturing mutation‐induced changes in binding affinity using limited labeled data. Large‐scale pre‐training equips DeepMutDTA with general DTA‐relevant representations, while SimSiam‐MuTF further refines these features by injecting mutation‐sensitive supervision in a data‐efficient manner. Across comprehensive benchmarks, DeepMutDTA‐MuTF achieves favorable performance against representative sequence‐based, protein language model‐based, and multimodal‐based DTA predictors, including under four challenging out‐of‐distribution settings, thereby suggesting the robustness and generalization potential of our approach.

Incorporating structural information, particularly pocket‐level geometry, is often employed to improve DTA prediction by providing mechanistic insights into molecular recognition [11, 12]. However, for mutation‐centric DTA tasks, structure‐driven pipelines face notable limitations. Labeled mutant interactions are frequently sparse, unevenly distributed, and biased toward specific targets, which can increase the risk of overfitting and unstable generalization when high‐dimensional structural features are utilized [17]. Despite the success of tools like AlphaFold2 in predicting wild‐type structures, accurately modeling mutants remains difficult and introduces extra uncertainty, especially when mutations trigger functionally significant conformational shifts [53]. These considerations motivated our emphasis on sequence‐based modeling, which bypasses the reliance on potentially noisy or unavailable mutant structures and aligns well with the sparse mutation–interaction regime. Notably, our empirical results support this design choice, with DeepMutDTA‐MuTF exhibiting competitive performance across multiple evaluation settings (Figure 2F–Q).

Within the sequence‐based paradigm, attention mechanisms have been shown to enhance predictive performance while offering interpretable insights into binding affinity [8, 9]. Given the substantial computational cost associated with large‐scale pre‐training, we adopted a top‐K attention strategy to balance expressiveness with efficiency. Importantly, mutation‐aware DTA prediction requires the capacity to distinguish between highly similar sequences that nonetheless exhibit substantial differences in binding affinity. Inspired by prior work [22, 23], we extended the SimSiam framework to supervised learning by incorporating task‐specific loss functions. This adaptation enables the embeddings learned by DeepMutDTA to align explicitly with binding affinity labels in both regression and classification contexts. Extensive experimental evaluations highlight the efficacy of SimSiam‐MuTF in mutation protein‐drug affinity prediction. Furthermore, case studies across diverse application scenarios indicate that DeepMutDTA can serve as a valuable computational resource for mutation‐aware protein–drug binding affinity evaluation, potentially supporting resistance monitoring and experimental hypothesis generation. Additionally, SimSiam‐MuTF serves as a plug‐and‐play fine‐tuning framework that not only boosts the performance of DeepMutDTA but also enhances other baseline methods. Its effectiveness extends even to the task of predicting mutational effects on protein‐protein interactions, highlighting its broad applicability.

Our study also points to broader challenges regarding the role of sequence similarity in the evaluation of protein machine learning models. In many protein‐centric tasks, such as protein function prediction, high sequence identity between training and test sets may artificially inflate performance by favoring similarity‐driven inference [54]. In contrast, predicting mutation‐induced changes in binding affinity appears to necessitate the modeling of fine‐grained functional effects that are not adequately captured by global sequence similarity alone. Even within a single protein background, varying the mutation site or introducing distinct substitutions at the same position can yield markedly different biophysical outcomes, including substantial affinity shifts and occasional conformational rearrangements [30]. This challenge closely parallels protein fitness prediction, which seeks to infer variant effects and remains difficult due to epistasis, nonlinear interactions, and limited coverage of the variant space [55, 56, 57]. Additionally, because even minor amino acid substitutions may induce complex and non‐linear changes in protein–drug interactions, precisely predicting the absolute binding affinity between a mutant protein and a drug remains challenging. Such absolute values may also be less directly actionable for wet‐lab experimental design. Therefore, assessing whether the predicted affinity changes are consistent with experimental trends, together with ranking potential resistance‐associated mutations, may provide a more practical and biologically informative alternative [58, 59, 60]. Consistent with this perspective, our performance evaluation and case studies suggest that DeepMutDTA‐MuTF can capture the consistency between predicted and observed affinity changes and can help prioritize potential resistance‐associated mutations for downstream validation.

Despite these encouraging results, several limitations deserve discussion. First, the current scope of our framework is primarily confined to the prioritization of key mutation profiles. Drug resistance, however, is governed by highly complex mechanisms, notably the epistatic interactions among co‐occurring mutations. Thus, while DeepMutDTA‐MuTF is highly effective as a predictive ranking tool, translating these rankings into a comprehensive understanding of the underlying structural and functional mechanisms remains a challenging task for future exploration. Second, although cold‐start evaluations indicate strong generalization potential, real‐world deployment will likely encounter more severe distribution shifts across both chemical space and target families. Incorporating uncertainty estimation, OOD detection, and selectively integrating lightweight multimodal signals, when reliable mutant structures or pocket annotations are available, may further enhance the framework's reliability for practical resistance surveillance and drug prioritization settings.

## 4 Experimental Section

### 4.1 Platinum Dataset

The Platinum dataset [61] is a manually curated, literature‐derived resource that focuses specifically on the impact of mutations on protein–drug binding affinity. It comprises over 1000 mutation entries and is the first dataset to comprehensively associate experimentally measured changes in binding affinity with the corresponding three‐dimensional structures of protein–drug complexes. We used protein–drug pairs as identifiers and ensured that there was no overlap between the pre‐training dataset and the Platinum dataset. After data cleaning, we obtained the corresponding PDB sequences and retrieved the drug SMILES representations from PubChem, resulting in a dataset comprising 981 mutations across 280 challenging protein–drug complexes.

For the Platinum dataset, we define two challenging tasks to evaluate the performance of DeepMutDTA. For the drug‐target binding affinity prediction task, we followed the prior work of DeepDTA [6] and normalized the labels to log space using the following formula:

(1) $$\text{pAffinity}=-\log_{10}\left(\frac{\text{Affinity}}{1e9}\right).$$

where Affinity denotes the protein–drug binding affinity, and can be $K_{i},K_{d}$, or ${\text{IC}}_{50}$.

We define the ΔAffinity prediction task as follows: Let ${\text{Affinity}}_{\text{WT}}$ and ${\text{Affinity}}_{\text{MT}}$ denote the binding affinities of the WT and MT forms of a given protein–drug pair, respectively. The ΔAffinity label is assigned based on the following criterion:

(2) $$\Delta \text{Affinity}=\left\{\begin{array}{cc} 0, & \text{if}\;{\text{pAffinity}}_{\text{WT}}>{\text{pAffinity}}_{\text{MT}}\\ 1, & \text{otherwise} \end{array}\right.$$

where ΔAffinity indicates the mutation direction: a label of 0 indicates that the mutation may confer resistance, whereas a label of 1 indicates that the mutation may confer sensitivity.

### 4.2 SARS‐CoV‐2 Dataset

The SARS‐CoV‐2 dataset [31] provides experimentally determined binding affinities between the SARS‐CoV‐2 main protease ($M^{\text{pro}}$) and three structurally distinct antiviral drugs: GC‐376, PF‐00835231, and PF‐07321332. In total, we curated 121 distinct drug‐target interaction pairs, consisting of 33 $M^{\text{pro}}$ variants interacting with GC‐376, 34 with PF‐00835231, and 54 with PF‐07321332.

### 4.3 Cancer Dataset

The Cancer dataset was compiled from COSMIC [62] and TTD [63] databases. Original data were downloaded from their respective web servers, subsequently integrated, and we ensured that there were no shared protein‐drug pairs among the pre‐training, fine‐tuning, and cancer datasets, resulting in a final dataset comprising 848 mutations distributed among 84 targets and 126 drugs. Based on the frequency distribution of mutations per target (Figure S12A), the dataset was partitioned into seven independent test sets, ensuring no overlap between the training/validation sets and test sets. Targets ABL1, FGFR2, EGFR, ALK, and AR were each considered individually as separate test sets due to their high mutation counts. Targets with mutation counts ranging between 20 and 40 were grouped into a combined dataset labeled Merge_20, while those targets with fewer than 20 mutations were consolidated into the Merge_10 dataset.

### 4.4 HIV Dataset

We collected literature‐documented protease inhibitor (PI) resistance records from the Stanford HIV Drug Resistance Database [64, 65]. This curation yielded 95 well‐characterized records detailing the interactions between the HIV‐1 protease and eight antiviral drugs.

### 4.5 Extended PPI Dataset

To verify the scalability of SimSiam‐MuTF, we incorporated two additional PPI mutation datasets: PPI_1102 [66] and PPI_1402 [67]. The PPI_1102 dataset focuses on single amino acid substitution in dimeric proteins and comprises 1102 interaction pairs across 57 proteins. The PPI_1402 dataset encompasses experimentally measured changes in binding affinity between wild‐type and mutated protein complexes, including single and multiple amino acid substitutions. It contains 1,402 interaction pairs for 114 proteins, with 1131 pairs involving single amino acid substitution, 195 pairs involving double‐point mutations, and 76 pairs involving three or more mutations.

### 4.6 Protein Sequence Encoding with FastFormer

We used FastFormer to generate an attention‐enhanced protein embedding matrix per sequence using the following steps [68]. First, given a protein sequence x of length L, we utilize the Tasks Assessing Protein Embeddings (TAPE) tokenizer [69] to map each residue into a discrete 25‐character alphabet. This vocabulary comprises 20 standard amino acids, 2 non‐standard amino acids (selenocysteine and pyrrolysine), 2 ambiguous, and 1 unknown amino acid. Subsequently, each residue is projected into a continuous feature space via a learnable embedding layer, yielding the initial sequence embedding $E_{p}^{init}=\left\{e_{1}^{init},e_{2}^{init},\text{…},e_{L}^{init}\right\}\in R^{L\times F}$, where L denotes the length of the protein sequence, and F represents the dimensionality of the feature space.

Next, linear transformations were applied to $E_{p}^{init}$ to generate the query (Q), key (K), and value (V) matrices:

(3) $$Q=E_{p}^{init}\cdot W_{q},\;K=E_{p}^{init}\cdot W_{k},\;V=E_{p}^{init}\cdot W_{v},$$

where $W_{q},W_{k},W_{v}\in R^{L\times L}$ are learnable weight matrices, and $Q=\{q_{1},\text{…},q_{L}\}$, $K=\{k_{1},\text{…},k_{L}\}$, $V=\{v_{1},\text{…},v_{L}\}$.

To efficiently capture the global sequence context without the computational bottleneck of standard self‐attention, FastFormer employs an additive attention mechanism. First, it computes an alignment score for each local query vector $q_{i}$ using a learnable global scoring weight $w_{\alpha }\in R^{F}$. These alignment scores are scaled and normalized via a softmax function to obtain the attention weights $\alpha_{i}$:

(4) $$\alpha_{i}=\frac{\exp(w_{\alpha }^{T}q_{i}/\sqrt{F})}{\sum_{j=1}^{L}\exp\left(w_{\alpha }^{T}q_{j}/\sqrt{F}\right)},$$

Using these normalized weights, the entire query matrix is then condensed into a single global query vector q through a weighted summation:

(5) $$q=\sum_{i=1}^{L}\alpha_{i}q_{i}.$$

Following the generation of the global query vector q, we model its interaction with each local key vector $k_{i}$ via an element‐wise product, yielding a set of interaction vectors $p_{i}=q*k_{i}$.

To aggregate these local interactions into a comprehensive global representation, we apply a second additive attention step. Specifically, we evaluate the importance of each interaction vector $p_{i}$ by computing its inner product with another learnable global scoring weight $w_{\beta }\in R^{F}$. These scores are normalized via a softmax function to produce the attention weights $\beta_{i}$:

(6) $$\beta_{i}=\frac{\exp(w_{\beta }^{T}p_{i}/\sqrt{F})}{\sum_{j=1}^{L}\exp\left(w_{\beta }^{T}p_{j}/\sqrt{F}\right)},$$

Subsequently, the interaction vectors are condensed into a single global context vector c through a weighted summation:

(7) $$c=\sum_{i=1}^{L}\beta_{i}p_{i}.$$

Finally, we perform an element‐wise product between the global context vector c and each local value vector $v_{i}$ to yield the key‐value interaction vector $u_{i}=c*v_{i}$.

Inspired by the vanilla Transformer [70], a linear projection layer is applied to these interaction vectors to map them into the final output space. To facilitate gradient flow and feature reuse, the output of this projection is added back to the corresponding original local query vector $q_{i}$ via a residual connection. Formally, the final attention‐enhanced representation $e_{i}^{p}$ for the i‐th residue is computed as:

(8) $$e_{i}^{p}=q_{i}+u_{i}\cdot W_{o}.$$

where $W_{o}\in R^{F\times F}$ is the learnable weight matrix of the output linear projection layer. By aggregating these updated token embeddings across the sequence, we obtain the final attention‐enhanced protein embedding matrix $E_{p}=\left[e_{1}^{p},e_{2}^{p},\text{…},e_{L}^{p}\right]$, where $E_{p}\in R^{L\times F}$.

### 4.7 Drug SMILES Encoding with MolFormer

Concurrently with extracting the amino acid embeddings of protein sequences via FastFormer, we utilize MolFormer [26] to encode the drug SMILES strings into token‐level representations. MolFormer is a large‐scale, pre‐trained chemical language model that employs a domain‐specific tokenizer [71] to parse SMILES strings into discrete tokens. Subsequently, its linear attention‐based Transformer architecture processes these tokens to generate initial embeddings with a hidden dimension of 768. To align the feature spaces for subsequent cross‐modal interactions, we apply a linear projection layer to map these 768‐dimensional embeddings into a target dimension F, matching that of the protein embedding. Formally, the final drug representation is denoted as $E_{d}\in R^{M\times F}$, where M represents the length of the drug token sequence.

### 4.8 Top‐K Attention and Importance Weighting for Drug–Target Interactions

Following initial feature extraction, modeling the intricate cross‐modal interactions between drug tokens and protein residues is often essential for estimating binding affinity. While standard dot‐product attention has the capacity to capture these pairwise relations, it computes similarities across all possible query‐key pairs. Such dense operations can incur substantial computational overhead and potentially introduce noise from weak, non‐informative interactions.

To mitigate this limitation, we implement a sparse Top‐k cross‐attention mechanism [72], which is hypothesized to isolate the most informative binding signals. Given the initial protein embeddings $E_{p}\in R^{L\times F}$ and drug embeddings $E_{d}\in R^{M\times F}$, we formulate two distinct cross‐attention branches: one focusing on the drug features contextualized by the protein, and the other on the protein features contextualized by the drug.

For the drug‐to‐protein attention branch, the queries are derived from the drug embeddings, while the keys and values are projected from the protein embeddings:

(9) $$Q^{d}=E_{d}W_{q}^{d},\;K^{p}=E_{p}W_{k}^{p},\;V^{p}=E_{p}W_{v}^{p},$$

where $W_{q}^{d},W_{k}^{p},W_{v}^{p}\in R^{F\times F}$ are learnable projection matrices. We compute the interaction scores but strictly retain only the k highest values for each query, masking the remaining entries. The attention‐enhanced drug representation $H^{d}\in R^{M\times F}$ is thus defined as:

(10) $$H^{d}=\text{softmax}\left(\text{Top-}k\left(Q^{d}{\left(K^{p}\right)}^{T}\right)\right)V^{p}.$$

Symmetrically, for the protein‐to‐drug attention branch, the protein embeddings serve as queries to extract relevant contextual features from the drug keys and values:

(11) $$Q^{p}=E_{p}W_{q}^{p},\;K^{d}=E_{d}W_{k}^{d},\;V^{d}=E_{d}W_{v}^{d},$$

(12) $$H^{p}=\text{softmax}\left(\text{Top-}k\left(Q^{p}{\left(K^{d}\right)}^{T}\right)\right)V^{d},$$

yielding the enhanced protein representation $H^{p}\in R^{L\times F}$.

Given these cross‐modal representations $H^{d}=\left[h_{1}^{d},\cdots ,h_{M}^{d}\right]$ and $H^{p}=\left[h_{1}^{p},\cdots ,h_{L}^{p}\right]$, we employ a modality‐specific attention pooling mechanism to quantify the predictive contribution of each position. For the i‐th drug token $h_{i}^{d}\in R^{F\times 1}$ and the j‐th protein residue $h_{j}^{p}\in R^{F\times 1}$, their unnormalized importance scores ($s_{i}^{d}$ and $s_{j}^{p}$) are computed via a two‐layer non‐linear transformation:

(13) $$s_{i}^{d}=W_{2}^{d}\tanh\left(W_{1}^{d}h_{i}^{d}+b_{1}^{d}\right)+b_{2}^{d},$$

(14) $$s_{j}^{p}=W_{2}^{p}\tanh\left(W_{1}^{p}h_{j}^{p}+b_{1}^{p}\right)+b_{2}^{p},$$

where $W_{1}^{m}\in R^{F_{attn}\times F}$, $W_{2}^{m}\in R^{1\times F_{attn}}$, $b_{1}^{m}\in R^{F_{attn}\times 1}$, and $b_{2}^{m}\in R$ denote the learnable weights and biases for each modality m∈{d,p}, and $F_{attn}$ indicates the hidden dimension of the pooling layer.

Subsequently, these scores are normalized across their respective sequence dimensions using a softmax function to yield the alignment weights:

(15) $$\omega_{i}^{d}=\frac{\exp(s_{i}^{d})}{\sum_{t=1}^{M}\exp\left(s_{t}^{d}\right)+\varepsilon },\;\omega_{j}^{p}=\frac{\exp(s_{j}^{p})}{\sum_{t=1}^{L}\exp\left(s_{t}^{p}\right)+\varepsilon },$$

with $\varepsilon =1\times 10^{-8}$ included to maintain numerical stability. A higher weight value suggests that the corresponding token or residue potentially holds greater discriminative power for the final affinity estimation.

The global sequence‐level representations are then derived through a weighted sum of the enhanced features:

(16) $$z_{d}=\sum_{i=1}^{M}\omega_{i}^{d}h_{i}^{d},\;z_{p}=\sum_{j=1}^{L}\omega_{j}^{p}h_{j}^{p},$$

resulting in the condensed, d‐dimensional embeddings for the drug ($z_{d}$) and the protein ($z_{p}$).

Ultimately, these global representations are concatenated and fed into a two‐layer multi‐layer perceptron (MLP) to predict the binding affinity. Formally, the predicted affinity score $\hat{y}$ is formulated as:

(17) $$E=z_{d}∥z_{p},\;\hat{y}=\text{MLP}\left(E\right)$$

where ∥ denotes the feature concatenation operation, and E represents the resulting drug‐target fusion representation.

### 4.9 Pre‐Training Strategy for DeepMutDTA

Following the cross‐modal interaction module, the protein sequences and drug SMILES strings are effectively integrated into fused drug‐target representations. Ultimately, to optimize the network's ability to decode the impact of mutations on drug binding, we adopt a two‐stage pre‐training and fine‐tuning paradigm. During the pre‐training stage, we leverage large‐scale drug‐target binding affinity datasets to endow DeepMutDTA with rich prior knowledge of molecular interactions. By formulating this pre‐training objective as a regression task, the model is directly optimized using the Mean Squared Error (MSE) loss function:

(18) $$L_{MSE}=\frac{1}{N}\sum_{i=1}^{N}{\left(y_{i}-{\hat{y}}_{i}\right)}^{2}.$$

where N represents the total number of training samples, while $y_{i}$ and ${\hat{y}}_{i}$ denote the ground‐truth and predicted binding affinity values for the i‐th sample, respectively.

### 4.10 Fine‐Tuning DeepMutDTA with SimSiam‐MuTF

Following pre‐training, it is generally necessary to fine‐tune the DeepMutDTA model using experimental mutation data. This step aims to better capture the potential shifts in binding affinity induced by single amino acid substitutions. To facilitate this, we propose a label‐aware contrastive fine‐tuning framework, termed SimSiam‐MuTF, inspired by the SimSiam architecture [22]. Specifically, SimSiam‐MuTF utilizes a Siamese network consisting of two weight‐sharing encoders, denoted as f (initialized with our pre‐trained DeepMutDTA weights), and a predictor head, denoted as h, which is designed to map the output of one view toward the representation of the other. During the fine‐tuning phase, the DeepMutDTA encoder and the predictor are optimized simultaneously. Following this training process, the predictor head is discarded, and the optimized DeepMutDTA (DeepMutDTA‐MuTF) is retained for downstream evaluation.

In practice, wild‐type (WT) sequences and their corresponding mutant (MT) sequences, paired with the same drug, are fed into the SimSiam‐MuTF architecture. The encoder yields embeddings $E_{1}^{WT}$ and $E_{2}^{WT}$, which serve as two distinct views for the WT sequence‐drug pairs, and similarly, $E_{1}^{MT}$ and $E_{2}^{MT}$ for the MT sequence‐drug pairs. Assuming the experimental binding affinities for these pairs are denoted as $y^{WT}$ and $y^{MT}$ respectively, and utilizing an MLP as the predictor head h, we formulate the initial fine‐tuning objective as:

(19) $$\begin{array}{c} \begin{array}{cc} L_{\text{DeepMutDTA}}= & \frac{1}{2}L\left(E_{1}^{WT}∥E_{2}^{MT},y^{WT}∥y^{MT}\right)\\ & +\frac{1}{2}L\left(E_{2}^{WT}∥E_{1}^{MT},y^{WT}∥y^{MT}\right), \end{array} \end{array}$$

where ∥ denotes the concatenation operation, and L(·) represents the task‐specific loss function.

In Siamese networks, omitting negative sample pairs may increase the risk of representational collapse. To mitigate this potential issue, we incorporate a stop‐gradient (stopgrad) mechanism, following the strategy proposed in the original SimSiam network [22]. Accordingly, the final objective function is modified to restrict gradient propagation through the target branch:

(20) $$\begin{array}{cc} L_{\text{DeepMutDTA}}= & \frac{1}{2}L\left(E_{1}^{WT}∥\text{stopgrad}\left(E_{2}^{MT}\right),y^{WT}∥y^{MT}\right)\\ & +\frac{1}{2}L\left(E_{2}^{WT}∥\text{stopgrad}\left(E_{1}^{MT}\right),y^{WT}∥y^{MT}\right). \end{array}$$

### 4.11 The Loss Function Designed For Regression Task

Drawing inspiration from [73], we employ a Rank‐N‐Contrast (RnC) loss in conjunction with the standard Mean Squared Error (MSE) loss. This joint optimization strategy guides the encoder to capture continuous variations across input data pairs. Specifically, the RnC loss extends contrastive learning to regression tasks by encouraging the distances in the learned embedding space to reflect the metric distances in the continuous label space.

For an anchor sample i and a reference sample j, we define a set of indices $S_{i,j}$ representing samples whose labels are further from the anchor than the reference label is:

(21) $$S_{i,j}=\left\{k|k\neq i,d\left(y_{i},y_{k}\right)>d\left(y_{i},y_{j}\right)\right\},$$

where d(·,·) denotes a distance metric (e.g., $L_{1}$ distance) in the label space. For a batch of 2N augmented samples, the overall RnC loss is formulated as:

(22) $$\begin{array}{c} L_{RnC}=\frac{1}{2N}\sum_{i=1}^{2N}\frac{1}{2N-1}\sum_{j=1,j\neq i}^{2N}-\log\frac{\exp(\text{sim}\left(E_{i},E_{j}\right)/\tau_{reg})}{\sum_{k\in S_{i,j}}\exp\left(\text{sim}\left(E_{i},E_{k}\right)/\tau_{reg}\right)}, \end{array}$$

where sim(·,·) represents the similarity measure between two feature embeddings (such as the negative $L_{2}$ norm), and $\tau_{reg}$ acts as a temperature hyperparameter.

Concurrently, to directly supervise the absolute binding affinity predictions, we compute the MSE loss over the same batch:

(23) $$L_{MSE}=\frac{1}{2N}\sum_{i=1}^{2N}{\left(y_{i}-\text{MLP}\left(E_{i}\right)\right)}^{2},$$

Ultimately, the overall objective function for the regression task is defined as a weighted sum of these two components:

(24) $$L_{reg}=\gamma L_{RnC}+\delta L_{MSE}.$$

where γ and δ are tunable hyperparameters utilized to balance the contrastive representation learning and the supervised regression.

### 4.12 The Loss Function Designed For Classification Task

Inspired by [74], we employ a combination of Supervised Contrastive Learning (SCL) and Binary Cross‐Entropy (BCE) loss functions to jointly optimize the pre‐trained DeepMutDTA model. For the classification task, the SimSiam‐MuTF framework leverages SCL to pull together the embeddings of protein‐drug pairs sharing the same interaction label, while effectively pushing apart the representations of distinct classes (e.g., distinguishing a binding wild‐type from a non‐binding mutant). The SCL loss across a batch of 2N samples is formulated as:

(25) $$\begin{array}{c} \begin{array}{ccc} L_{SCL} & = & \sum_{i=1}^{2N}\frac{-1}{N_{y_{i}}-1}\sum_{j=1}^{2N}1_{\left[i\neq j\right]}1_{\left[y_{i}=y_{j}\right]}\log\\ & & \frac{\exp(\text{sim}\left(Z_{i},Z_{j}\right)/\tau_{cls})}{\sum_{k=1}^{2N}1_{\left[i\neq k\right]}\exp\left(\text{sim}\left(Z_{i},Z_{k}\right)/\tau_{cls}\right)}, \end{array} \end{array}$$

where $Z_{i}=f\left(E_{i}\right)$ represents the projected embedding, $N_{y_{i}}$ denotes the total number of samples in the batch sharing the same label $y_{i}$, $1_{\left[\cdot \right]}$ is an indicator function evaluating to 1 if the condition is true and 0 otherwise, and τ is the temperature parameter.

Concurrently, the standard BCE loss is applied to provide direct supervision for the classification probabilities:

(26) $$L_{BCE}=-\frac{1}{2N}\sum_{i=1}^{2N}\left[y_{i}\log\left({\hat{y}}_{i}\right)+\left(1-y_{i}\right)\log\left(1-{\hat{y}}_{i}\right)\right],$$

where ${\hat{y}}_{i}=\text{MLP}\left(E_{i}\right)$ denotes the predicted probability for the i‐th sample.

The overall objective for the classification task is defined as the weighted sum of these two losses:

(27) $$L_{cls}=\gamma L_{SCL}+\delta L_{BCE}.$$

with hyperparameters γ and δ controlling the relative contributions of the contrastive and classification objectives.

### 4.13 Implementation Details and Running Time

The entire model architecture is trained in an end‐to‐end manner, with all parameters updated continuously during the process. Specifically, the Molformer module is initialized with pre‐trained weights but is fully fine‐tuned alongside the other components. Conversely, the Fastformer module and all Multi‐Layer Perceptrons are initialized randomly and trained from scratch. In the pre‐training stage, the learning rate was set to 0.0001, the batch size was set to 100, and the epochs were set to 40. During the cross‐attention phase, we selected Top‐K as 32. We set the limitation of protein sequence to 1000, and the length of the drug SMILES token is 100. The models were optimized using the Adam optimizer and trained on an NVIDIA RTX 4090 GPU (24GB) for accelerated processing. The DeepMutDTA model was trained for one epoch ∼40 minutes. In the fine‐tuning stage, we set the learning rate to 5e‐5 and the number of epochs to 500. We defined the hyperparameter search space for γ and δ over the interval [0.1, 0.9], and the optimal hyperparameter values are listed in Table S9 (Supporting Information).

### 4.14 Molecular Dynamics Simulations

All molecular dynamics simulations were carried out in GROMACS 2021.7, with ligand parameters generated via the CGenFF. The protein–ligand complex was solvated in a cubic box with a 1.0 nm buffer of TIP3P water and neutralized to 0.15 M NaCl. After steepest‐descent energy minimization (50,000 steps), the system underwent 100 ps of NVT equilibration at 300 K and 100 ps of NPT equilibration at 1 bar, both with positional restraints on heavy atoms. Finally, a 100‐ns unrestrained production run was performed in the NPT ensemble, and trajectory analyses (RMSD and Coul‐SR) were conducted using built‐in GROMACS tools.

### 4.15 Statistical Analysis

Continuous data are presented as mean ± standard deviation (SD). Linear regressions were assessed by Pearson's correlation coefficient (PCC) and Spearman's correlation coefficient (SCC). For statistical comparisons of our method against competing approaches across all evaluation settings, we evaluated the results at a 95% confidence level. A one‐way ANOVA was used to assess significance. In all cases, significance was defined as *p* < 0.05. All statistical testing was done in Python 3.8.19 using the scipy package (v1.10.1).

## Author Contributions

X.H. and P.Z. contributed equally to this work. X.H., P.Z., L.D. and Z.S. performed in conceptualization. X.H. and P.Z. performed in data curation. X.H., P.Z. and S.W. performed in methodology. X.H., P.Z. and S.W. performed in investigation. X.H., H.S., M.L., L.D. and Z.S. performed in visualization. X.H., L.D. and Z.S. performed in writing – original draft. X.H., P.Z., Z.S., S.S. and L.D. performed in writing – review & editing. L.D. and Z.S. performed in supervision. L.D. performed in funding acquisition.

## Funding

This research was supported by the National Natural Science Foundation of China (Grant Nos. U23A20321 and 62272490) and the Natural Science Foundation of Hunan Province of China (Grant No. 2025JJ20062).

## Conflicts of Interest

The authors declare no conflicts of interest.

## Supporting information


**Supporting File**: advs76831‐sup‐0001‐SuppMat.pdf.

## Data Availability

DeepMutDTA is open‐source software distributed under the MIT license and is available on GitHub (https://github.com/altriavin/DeepMutDTA). The BindingDB dataset can be accessed at https://www.bindingdb.org/rwd/bind/index.jsp, while the BioLip dataset is available at https://zhanggroup.org/BioLiP/. Additionally, the Platinum dataset can be found at https://biosig.lab.uq.edu.au//. The SARS‐CoV‐2 database can be available at https://github.com/altriavin/DeepMutDTA. PPI_1102 and PPI_1402 databases can be available at https://github.com/altriavin/DeepMutDTA. The HIV dataset can be available at https://hivdb.stanford.edu/dr‐summary/resistance‐notes/PI/. The TTD dataset can be available at https://idrblab.net/ttd/. The COSMIC dataset can be available at https://cancer.sanger.ac.uk/cosmic. The GISAID dataset can be downloaded from https://gisaid.org/. Source code can be available on https://github.com/altriavin/DeepMutDTA.
