# Supplementary material for: Deep Contrastive Learning for High‐Throughput Prediction of Drug Resistance Mutations from Sequences
Source: Adv Sci (Weinh). 2026 Jul 29:e14899. Online ahead of print. doi: 10.1002/advs.202514899 (PMC13418287; doi:10.1002/advs.202514899)
Supplement: Supplementary file 1 — Supporting File: advs76831‐sup‐0001‐SuppMat.pdf. [file ADVS-9999-e14899-s001.pdf]

Supplementary Information for Deep Contrastive Learning for High-Throughput Prediction of Drug Resistance Mutations from Sequences

Xiaowen Hu[1†], Pan Zhang[2, 3†], Shangqian Wu[1], Hao Sun[1], Minwei Li[1], Sophia Tsoka[4], Zizhang Sheng[5*], Lei Deng[1*]

[1]School of Computer Science and Engineering, Central South University, Changsha, 410083, Hunan, China.
[2]Infection Control Center, Xiangya Hospital of Central South University, Changsha, 410008, Hunan, China.
[3]National Clinical Research Center for Geriatric Disorders, Xiangya Hospital of Central South University, Changsha, 410008, Hunan, China.
[4]Department of Informatics, King's College London, London, WC2B 4BG, United Kingdom.
[5]Aaron Diamond AIDS Research Center, Columbia University Vagelos College of Physicians and Surgeons, New York, 10032, NY, United States.

*Corresponding author(s). E-mail(s): zs2248@cumc.columbia.edu; leideng@csu.edu.cn;
†These authors contributed equally to this work.

Supplementary Notes

S1. Performance Evaluation and Interpretability Analysis of Pre-trained DeepMutDTA

To evaluate the efficacy of DeepMutDTA, we conducted a comparative analysis against three well-established DTA prediction models using the Pre-Training dataset (detailed in the Pre-Training Dataset section): DeepDTA [1], AttentionDTA [2], and HyperAttentionDTI [3]. We utilized the Pearson correlation coefficient (PCC) and the Spearman correlation coefficient (SCC) as evaluation metrics. As illustrated in Fig. S1A, DeepMutDTA consistently outperformed the baseline methods, achieving an average PCC of 0.842 and an average SCC of 0.841. Specifically, DeepMutDTA demonstrated notable improvements of 4.59% in PCC and 4.60% in SCC relative to the second-best performing model. Statistical analysis via ANOVA on the five-fold cross-validation results confirmed the significance of these improvements, with all corresponding p-values below 0.05.

We further assessed the generalizability of DeepMutDTA to unseen targets, a critical indicator of its potential for real-world applications. To this end, we evaluated the model using two external benchmark datasets (detailed in the Virtual Screening Dataset section). The performance of the pre-trained model was quantified by the area under the receiver operating characteristic curve (AUC), Boltzmann-Enhanced Discrimination of ROC (BEDROC), and enrichment factor (EF). On the DUD-E dataset (Fig. S1B), comprising 102 targets along with corresponding decoy molecules, DeepMutDTA achieved an average AUC of 0.6792, outperforming HyperAttentionDTI (average AUC = 0.6402). Similarly, on the DEKOIS2.0 dataset (Fig. S1C), DeepMutDTA attained an average AUC of 0.8186, representing a substantial 13.8% improvement relative to the next-best model. Moreover, DeepMutDTA consistently outperformed all baseline methods in terms of both BEDROC and EF metrics across both benchmark datasets (Figs. S2 and S3). To further investigate the feature representations learned by each model, we selected two representative targets from each benchmark dataset: WEE1 and VGFR2 from DUD-E, and BRAF and AKT1 from DEKOIS2.0. The embeddings of drug-target pairs generated by each model were visualized using t-SNE. The feature distributions learned by the pre-trained DeepMutDTA are illustrated in Figs. S1D-G, whereas the corresponding visualizations for DeepDTA, AttentionDTA, and HyperAttentionDTI are provided in Fig. S4. Figs. S1D-G demonstrate that the pre-trained DeepMutDTA effectively discriminates between active compounds and decoys, leading to distinct clustering of these two groups.

To further evaluate DeepMutDTA's ability to identify critical drug-target binding sites, we applied the pre-trained model to predict binding-site residues for 102 protein targets from the DUD-E dataset using their Protein Data Bank (PDB) structures. By inputting the PDB-derived sequences along with the corresponding ligand SMILES representations into DeepMutDTA, we obtained importance scores for both protein residues and ligand tokens. These predictions were subsequently assessed using the Protein Contacts Atlas [4]. Detailed results regarding the number of correctly identified amino acids are shown in Fig. S5. Out of the 102 targets, 82 yielded at least

one correctly predicted binding site. Notably, Table S1 highlights eight specific examples where DeepMutDTA identified eight or more true binding-site residues within its top 20 predictions. To illustrate this in greater detail, we conducted case studies on two representative DUD-E targets: PA2GA (PDB ID: 1KVO [5]) and SRC (PDB ID: 3EL8 [6]). For PA2GA, DeepMutDTA successfully pinpointed 12 known binding-site residues within its top 20 predictions (L54, Y111, L19, E55, Y51, L2, L11, L87, S20, Y105, Y21, and Y24). A three-dimensional visualization of these modeled drug-target interactions, generated using PLIP [7], is presented in Fig. S1H. Similarly, for SRC, the model captured nine critical binding residues within its top 20 predictions, namely K316, K321, V271, Q324, K401, K295, Y340, K343, and Y376. These interactions are depicted in Fig. S1I. Collectively, these findings suggest that DeepMutDTA can effectively highlight biologically relevant drug-target binding sites, thereby supporting its interpretability and demonstrating its potential generalizability to previously unseen targets.

S2. Pre-Training Dataset

For the Pre-training dataset, we compiled relevant IC50 data from BindingDB[8] and BioLip[9] to construct a comprehensive benchmark dataset. To ensure data quality and reliability, we filtered out invalid SMILES representations, resulting in a final dataset containing 1,459,913 interactions between 798,390 unique drugs and 16,202 protein sequences. And then, we followed the prior work of DeepDTA[1] to change the Affinity value into the log space as follows:

$$\text{pAffinity} = -\log_{10}\left(\frac{\text{Affinity}}{1e9}\right). \tag{1}$$

where Affinity denotes the protein–ligand binding affinity. We employed a five-fold cross-validation strategy to train and evaluate DeepMutDTA, using the mean and standard deviation of the results from the five folds as the final evaluation metrics.

S3. Virtual Screening Dataset

For our virtual screening experiments, we selected two benchmark datasets: DUD-E[10] and DEKOIS2.0[11]. The DUD-E dataset contains 22,886 positive ligands targeting 102 proteins from various families, including GPCRs, kinases, nuclear receptors, proteases, ion channels, cytochrome P450s, enzymes, and other miscellaneous proteins. These ligands were originally sourced from the ChEMBL database[12]. Each positive ligand was complemented with 50 decoys generated from the ZINC database[13], ensuring similar physicochemical properties but distinct 2D topologies. The DEKOIS2.0 dataset consists of 81 targets, each associated with 40 positive compounds obtained from BindingDB[8] and 1,200 decoys created using ZINC.

S4. OOD Scenarios Dataset Split

We established four train-test split configurations to assess prediction performance:

1. Regime Scenario: This scenario explores how mutations combine by training on single amino acid substitutions and predicting the effects of multiple substitutions.
2. Cold Drug Scenario: In this scenario, 20% of the drugs were completely withheld as the test set. This simulates a drug repositioning situation where the model predicts previously unseen drugs.
3. Cold Protein Scenario: Here, 20% of the proteins were entirely withheld as the test set. This simulates a drug repositioning scenario in which the model predicts previously unseen proteins.
4. Cold Both Scenario: In this case, 20% of both proteins and drugs were completely withheld, creating a test set that simulates the circumstance where the model must predict both unseen proteins and drugs.
5. Sequence Identity Split: This setting is related to the cold-protein split but is more stringent: it not only ensures that proteins in the training and test sets are different, but also enforces that the maximum pairwise sequence identity between any training protein and any test protein is bounded by a threshold T, where $T \in \{10\%, 20\%, 30\%, 40\%, 50\%\}$. To construct this split, we used SpanSeq[14] to cluster protein sequences under the corresponding identity threshold T. We then assigned entire clusters to either the training or the test set. Clusters were sampled and added to the split until the desired set size was reached. This cluster-wise assignment constrains cross-set similarity and yields a controlled, progressively harder out-of-distribution evaluation as T decreases.

Supplementary Figures

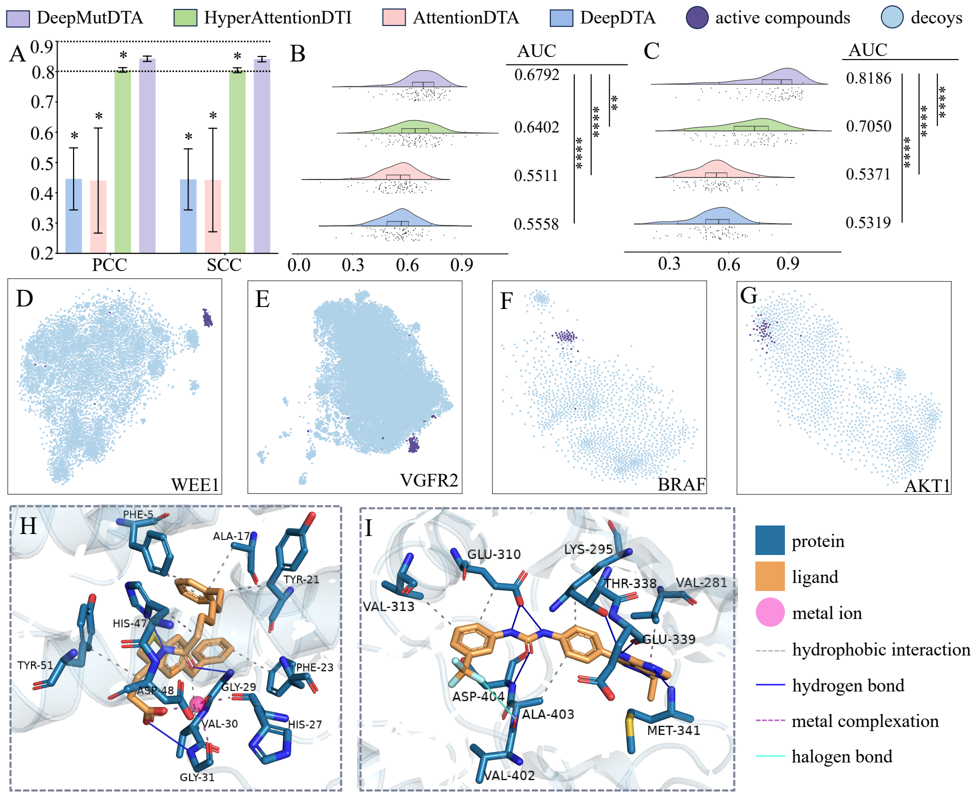

Fig. S1 Comparative performance and feature analysis of DeepMutDTA and baseline DTA models. (A) Predictive performance comparison of DeepMutDTA, DeepDTA, AttentionDTA, and HyperAttentionDTI evaluated using PCC and SCC on benchmark datasets. Statistical significance is indicated as follows: *p < .05; **p < .01; ***p < .001; ****p < .0001. (B and C) Cloud-rain plots illustrate the distribution of predicted AUC values across individual targets from the DUD-E (B) and DEKOIS2.0 (C) datasets. Each data point corresponds to the predicted AUC for a specific target, demonstrating the prediction variability among targets. (D to G) t-SNE visualizations of the feature embeddings generated by DeepMutDTA for representative targets: WEE1 (D) and VGFR2 (E) from the DUD-E dataset, and BRAF (F) and AKT1 (G) from the DEKOIS2.0 dataset. (H to I) Three-dimensional visualization of predicted drug-target interactions highlighting critical binding-site residues identified by DeepMutDTA for PA2GA (H) and SRC (I).

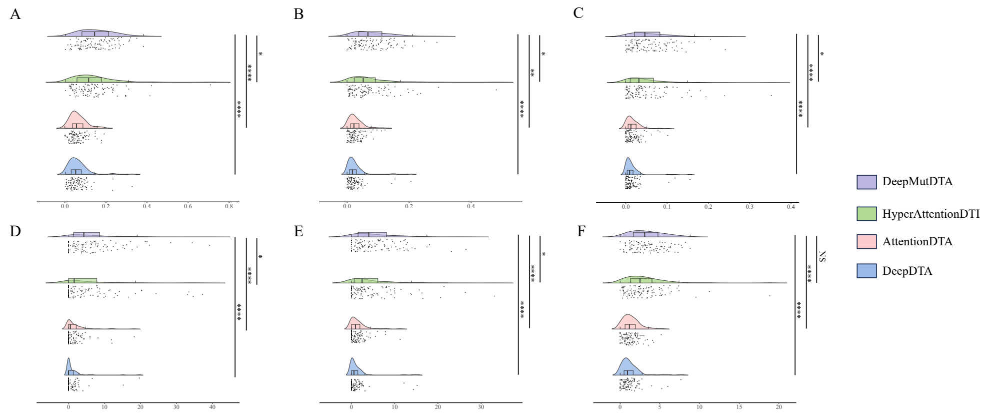

Fig. S2 Evaluation of five methods on DUD-E dataset. (A): BEDROC($\alpha = 20$), (B): BEDROC($\alpha = 50$), (C): BEDROC($\alpha = 80.5$), (D): 0.5% EF, (E): 1% EF, (F): 5% EF. * $p < .05$; ** $p < .01$; ***$p < .001$; ****$p < .0.0001$.

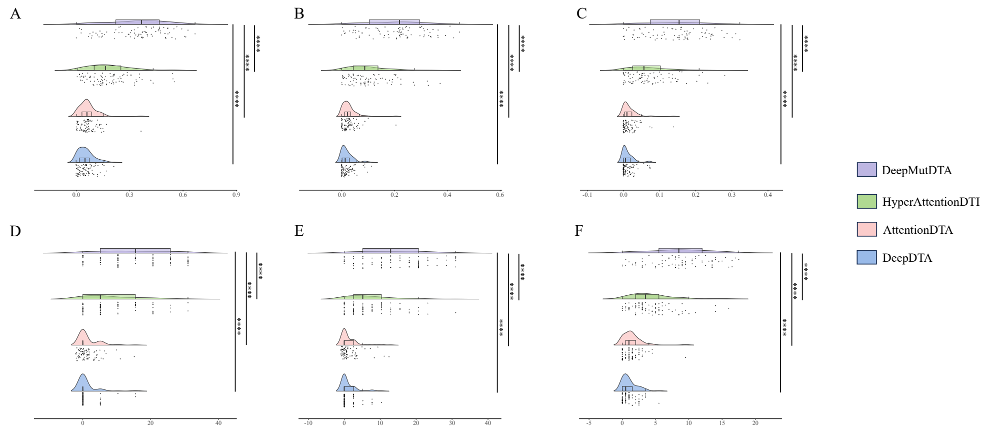

Fig. S3 Evaluation of five methods on DEKOIS2.0 dataset. (A): BEDROC($\alpha = 20$), (B): BEDROC($\alpha = 50$), (C): BEDROC($\alpha = 80.5$), (D): 0.5% EF, (E): 1% EF, (F): 5% EF. * $p < .05$; ** $p < .01$; ***$p < .001$; ****$p < .0.0001$.

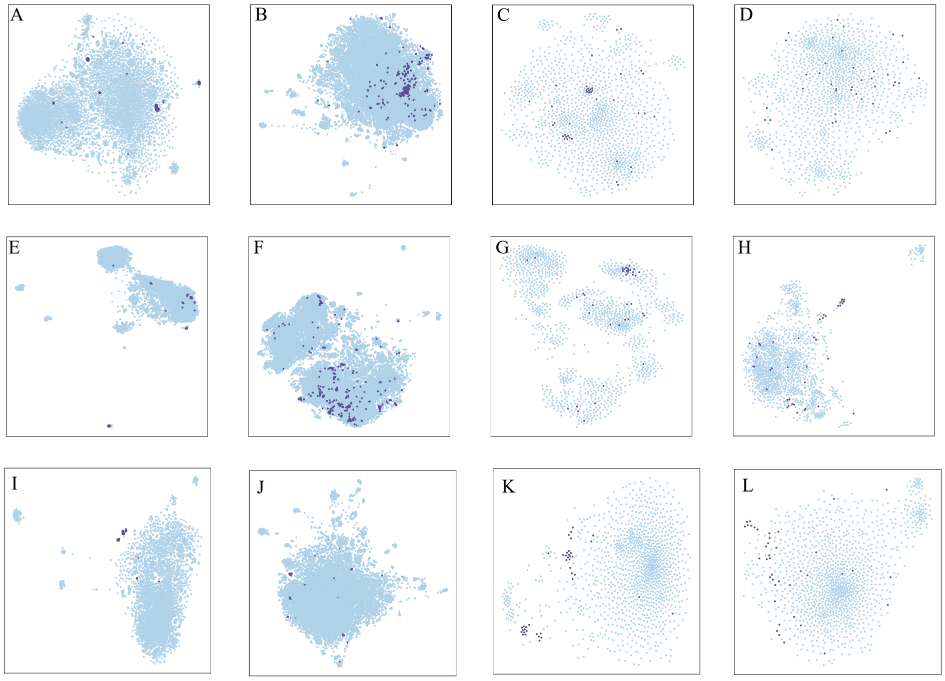

Fig. S4 t-SNE visualization results for three models (DeepDTA, AttentionDTA, and HyperAttentionDTI) across four targets (wee1, VGFR2, BRAF, and AKT1). (A to D): DeepDTA on wee1 (A), VGFR2 (B), BRAF (C), and AKT1 (D). (E to H): AttentionDTA on wee1 (E), VGFR2 (F), BRAF (G), and AKT1 (H). (I to L): HyperAttentionDTI on wee1 (I), VGFR2 (J), BRAF (K), and AKT1 (L).

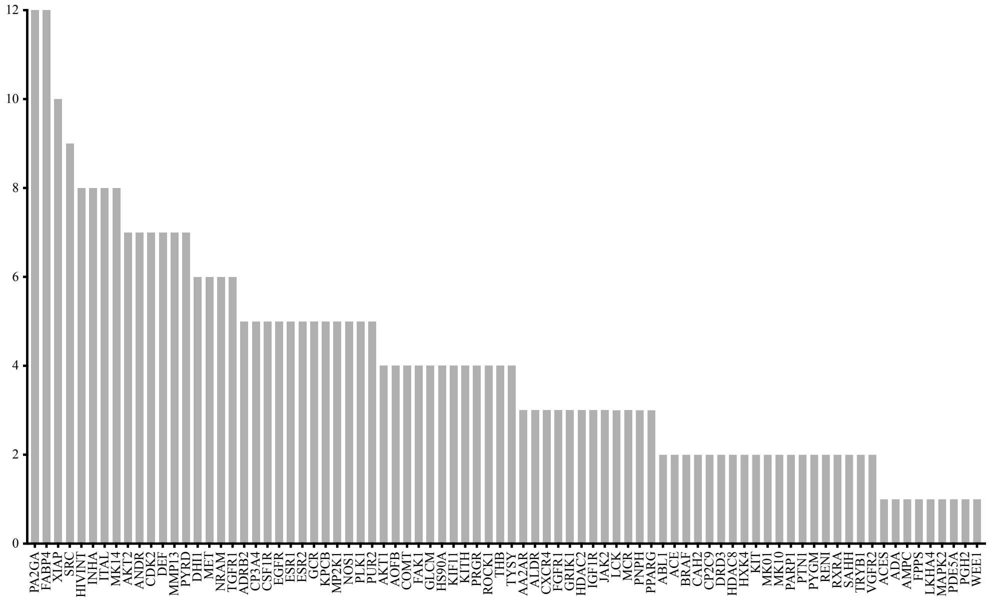

Fig. S5 Number of critical binding-site residues correctly identified by DeepMutDTA across DUD-E targets.

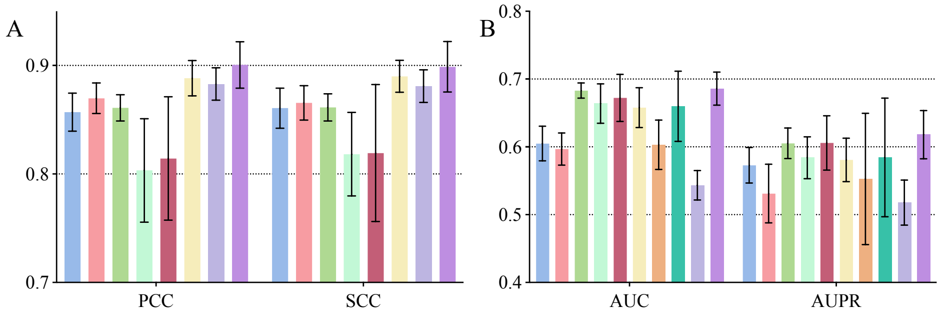

Fig. S6 The performance of 5-fold CV dataset split on binding affinity prediction (A) and ΔAffinity prediction task (B).

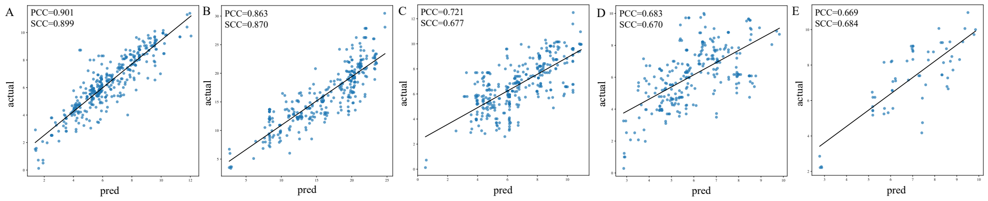

Fig. S7 The scatter plots of DeepMutDTA-MuTF. (A–E) Scatter plots of DeepMutDTA-MuTF under the 5CV, Regime, Cold Protein, Cold Drug, and Cold Both scenarios.

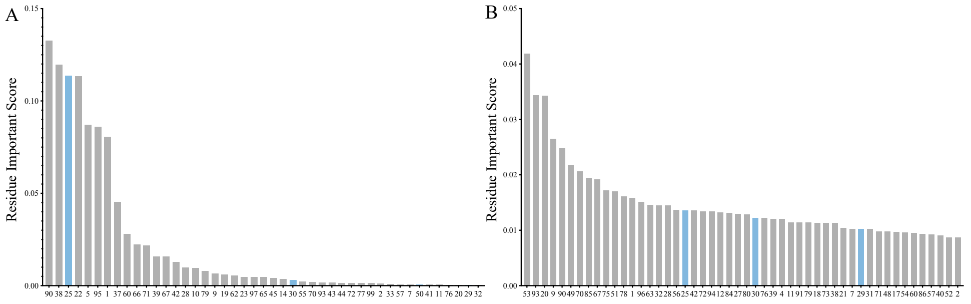

Fig. S8 A reverse x-axis bar chart displays the predicted residues that contribute to drug sensitivity based on the important scores from the DeepMutDTA-MuTF (A) and TransformerCPI2.0 (B) model for the HIV-1 WT sequence and Amprenavir. Blue highlights the causal sensitivity-conferring sites.

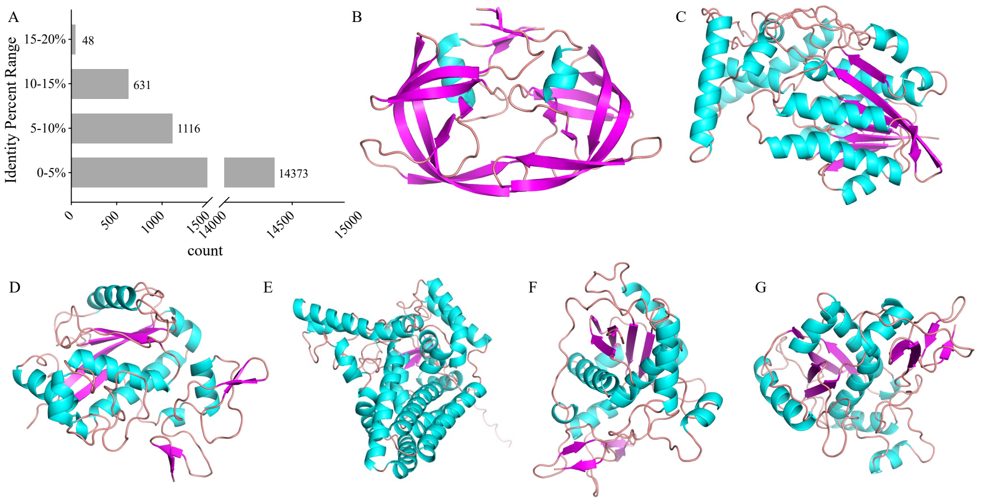

Fig. S9 Sequence-identity assessment of the refined pre-training dataset after removal of HIV-1 protease entries. (A) Distribution of sequence identity between HIV-1 protease and the 16,168 remaining unique proteins, calculated using Clustal Omega. (B) Reference HIV-1 protease sequence. (C–G) The five proteins with the highest sequence identity to HIV-1 protease among the refined dataset.

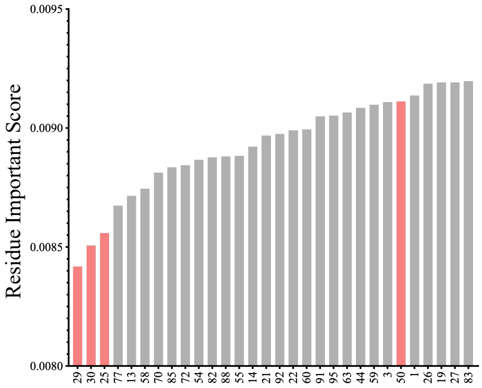

Fig. S10 A reversed bar chart showing the predicted resistance-contributing residues after HIV-1 protease entries were removed from the pre-training dataset, based on residue importance scores derived from DeepMutDTA-MuTF. Red bars indicate causal resistance-conferring sites.

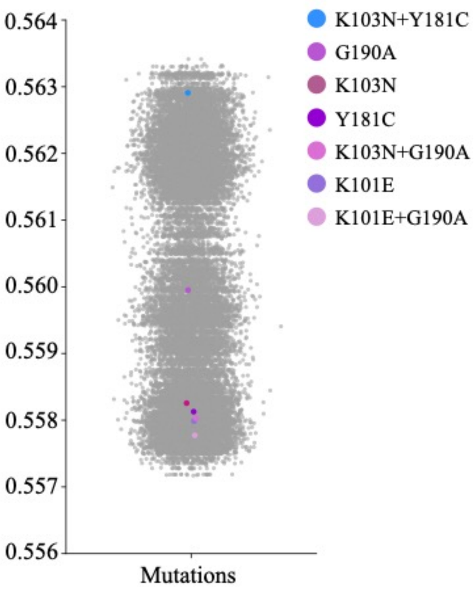

Fig. S11 Predicted resistance probability distribution for isolated and combined HIV-1 mutations.

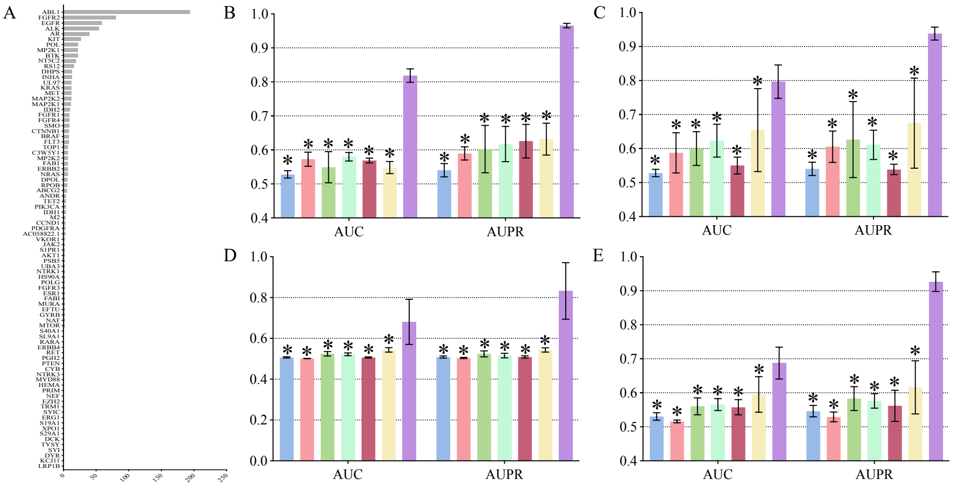

Fig. S12 Mutation Frequencies and Model Performance on ABL1, EGFR, and Merged Datasets. (A) The frequency distribution of mutations in each target. (B to E) the performance of model on ABL1 (B), EGFR (C), Merge_10 (D) and Merge_20 (E). *$p < .05$

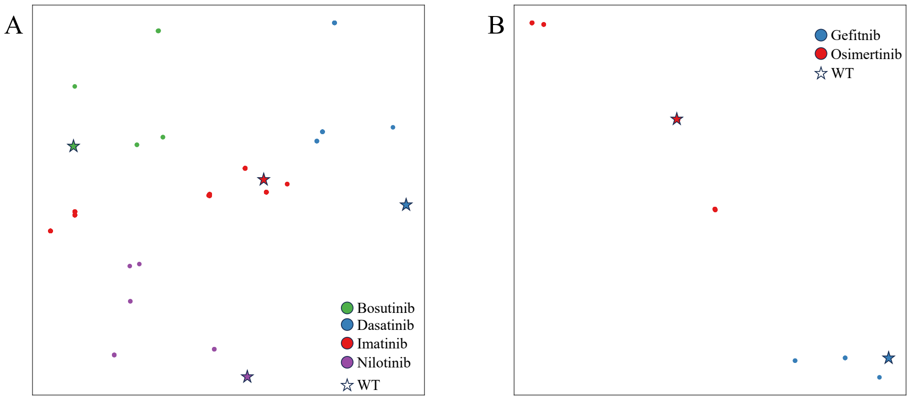

Fig. S13 t-SNE visualization results ABL1 (A) and EGFR (B).

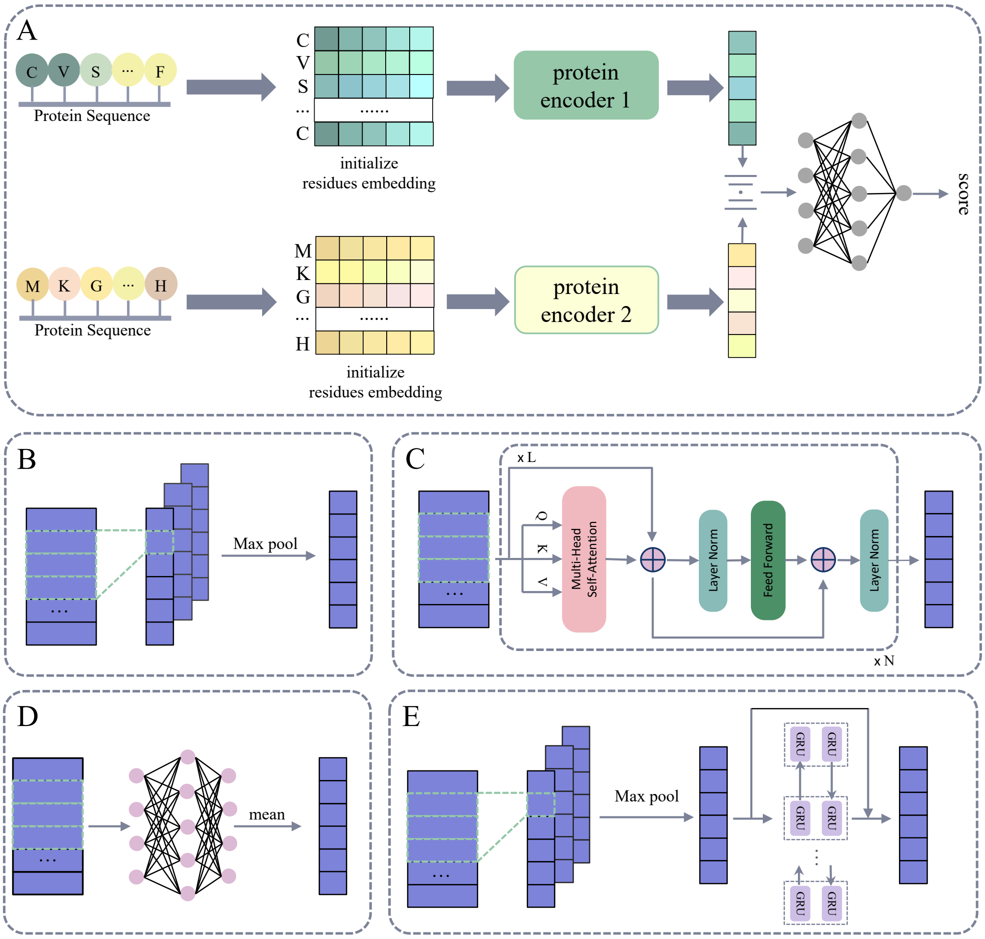

Fig. S14 Protein–Protein Interaction Model Overview. (A) the Protein-Protein Interaction (PPI) model. (B to E): Common encoder modules used in the models. B represents a Convolutional Neural Network (CNN), C shows a Transformer, D depicts a Deep Neural Network (DNN), and E features a Recurrent Convolutional Neural Network (RCNN).

Supplementary Tables

Table S1 Predicted key amino acid binding sites for representative DUD-E targets by DeepMutDTA.

Target Name	PDB ID	Hit Amino Acids
PA2GA	1KVO	L54, Y111, L19, E55, Y51, L2, L11, L87, S20, Y105, Y21, Y24
FABP4	2NNQ	E72, E14, K9, L91, E54, E61, E129, D17, V127, Q95, L110, G6
XIAP	3HL5	K322, K211, K297, V298, K299, L307, Y324, E318, S313, L284
SRC	3EL8	K316, K321, V271, Q324, K401, K295, Y340, K343, Y376
HIVINT	3NF7	A76, N155, K159, Q148, Q62, D64, Q146, K160
INHA	4TRJ	I122, W230, A164, W222, A260, V203, L44, A157
ITAL	2ICA	V171, S279, Y257, D156, S165, Q303, K155, K232
MKL4	2QD9	V117, A40, K53, A34, K54, V38, A41, S37

Table S2 The confusion matrix of the prediction result of DeepMutDTA-MuTF on the pocket site.

Pocket site	TP	FP	TN	FN	Pocket site	TP	FP	TN	FN
H41	0	3	17	0	H164	1	3	16	0
M49	0	19	1	0	E166	3	16	1	0
Y54	0	2	16	2	L167	1	15	4	0
F140	1	3	15	1	P168	0	1	19	0
L141	0	4	16	0	H172	0	11	9	0
N142	0	2	18	0	D187	0	1	16	3
G143	0	14	6	0	R188	0	17	2	1
S144	1	1	12	6	Q189	0	1	18	1
C145	0	1	19	0	T190	0	12	8	0
H163	0	19	1	0	Q192	10	9	0	1
M165	1	3	16	0	SUM	18	157	230	15

Table S3 The affinity of SARS-CoV-2 M^{pro} variants to nirmatrelvir, predicted by DeepMutDTA-MuTF

Mutation	Predicted Affinity
D229N	10.0451
A116S/L115F	9.4973
A193P	9.3802
A193P/P132H	9.2434
P132H/A7S	9.9435

Table S4 Top-20 mutations predicted to impair binding affinity, generated via in silico deep mutational scanning of the HIV-1 capsid against lenacapavir (LEN).

rank	variant	rank	variant	rank	variant	rank	variant	rank	variant
1	N74T	5	N74S	9	N74D	13	N74M	17	N74R
2	N74P	6	N74Q	10	N74Y	14	N74W	18	V181E
3	N74E	7	N74V	11	N74K	15	N74A	19	N74F
4	N74C	8	N74G	12	N74I	16	N74H	20	N74L

Table S5 Top-20 combined mutations predicted to exhibit potential epistatic effects and impair binding affinity.

rank	variant	rank	variant	rank	variant	rank	variant
1	L100V+K101G	6	K103E+G190I	11	K103E+G190H	16	K103E+G190T
2	L100T+K101G	7	K103E+G190S	12	K103E+G190Y	17	K103E+G190W
3	L100A+K101G	8	K103E+G190F	13	K103E+G190R	18	K103E+G190M
4	K103E+G190C	9	K103E+G190D	14	K103E+G190L	19	K103E+G190Q
5	K103E+G190V	10	K103E+G190P	15	K103E+G190N	20	K103E+G190K

Table S6 The result of ablation study
* For the regression task, the task-based loss function is the BCE loss function, while for classification, the task-based loss function is the MSE loss function.
# For the regression task, the task-specify loss function is the RnC loss function, while for classification, the task-specify loss function is the SCL loss function.

				regression		classification	
*	#	SimSiam-MuTF	Pretrain	PCC	SCC	AUC	AUPR
✓				0.839±0.031	0.838±0.031	0.707±0.033	0.759±0.044
✓			✓	0.843±0.007	0.840±0.014	0.710±0.077	0.747±0.080
✓		✓		0.830±0.032	0.831±0.025	0.720±0.036	0.719±0.024
✓		✓	✓	0.860±0.08	0.855±0.009	0.721±0.042	0.749±0.049
	✓			0.684±0.141	0.659±0.179	0.711±0.121	0.746±0.109
	✓		✓	0.727±0.029	0.675±0.052	0.737±0.045	0.801±0.050
	✓	✓		0.410±0.114	0.375±0.124	0.708±0.068	0.731±0.085
	✓	✓	✓	0.500±0.189	0.483±0.207	0.736±0.040	0.721±0.049
✓	✓			0.815±0.032	0.823±0.026	0.724±0.134	0.752±0.114
✓	✓		✓	0.839±0.015	0.835±0.014	0.743±0.035	0.754±0.046
✓	✓	✓		0.883±0.013	0.882±0.013	0.741±0.047	0.768±0.053
✓	✓	✓	✓	0.900±0.021	0.898±0.023	0.787±0.041	0.840±0.048

Table S7 The performance of finetuning the comparative methods using SimSiam-MuTF on the regression and classification task

Method	regression		classification	
	PCC	SCC	AUC	AUPR
DeepDTA	0.857 ± 0.017	0.860 ± 0.018	0.538 ± 0.031	0.536 ± 0.037
DeepDTA (SimSiam-MuTF)	0.868 ± 0.016	0.867 ± 0.015	0.543 ± 0.041	0.553 ± 0.058
Δ	1.28%	0.81%	0.93%	3.17%
AttentionDTA	0.869 ± 0.014	0.865 ± 0.015	0.510 ± 0.004	0.517 ± 0.010
AttentionDTA (SimSiam-MuTF)	0.886 ± 0.007	0.883 ± 0.013	0.586 ± 0.049	0.576 ± 0.068
Δ	1.96%	2.08%	14.9%	11.42%
HyperAttentionDTI	0.860 ± 0.012	0.861 ± 0.012	0.610 ± 0.077	0.619 ± 0.086
HyperAttentionDTI (SimSiam-MuTF)	0.892 ± 0.011	0.888 ± 0.012	0.674 ± 0.027	0.702 ± 0.031
Δ	3.72%	3.14%	10.49%	13.41%
MFE	0.803 ± 0.047	0.818 ± 0.038	0.655 ± 0.057	0.671 ± 0.048
MFE (SimSiam-MuTF)	0.817 ± 0.045	0.839 ± 0.024	0.657 ± 0.031	0.676 ± 0.052
Δ	1.74%	2.57%	0.31%	0.75%
DrugBAN	0.814 ± 0.056	0.819 ± 0.063	0.508 ± 0.002	0.509 ± 0.010
DrugBAN (SimSiam-MuTF)	0.820 ± 0.015	0.829 ± 0.017	0.515 ± 0.005	0.524 ± 0.006
Δ	0.74%	1.22%	1.38%	2.95%
TransformerCPI2.0	0.888 ± 0.016	0.890 ± 0.014	0.663 ± 0.097	0.704 ± 0.106
TransformerCPI2.0 (SimSiam-MuTF)	0.892 ± 0.022	0.898 ± 0.016	0.702 ± 0.027	0.763 ± 0.021
Δ	0.45%	0.90%	5.88%	8.38%

Table S8 Performance analysis of four encoding methods on two PPI datasets with and without SimSiam-MuTF

	PPI_1402		PPI_1102	
	PCC	SCC	PCC	SCC
DNN(MSE)	0.8750±0.0243	0.8399±0.0280	0.8383±0.0055	0.8129±0.0097
DNN(SimSiam-MuTF)	0.8926±0.0018	0.8498±0.0050	0.8483±0.0065	0.8282±0.0061
$\triangle$	1.97%	1.16%	1.17%	1.84%
CNN(MSE)	0.8822±0.0041	0.8481±0.0120	0.9336±0.0036	0.9270±0.0043
CNN(SimSiam-MuTF)	0.8945±0.0087	0.8694±0.0176	0.9406±0.0051	0.9357±0.0054
$\triangle$	1.37%	2.44%	0.74%	0.92%
RCNN(MSE)	0.9216±0.0082	0.8920±0.0091	0.9039±0.0150	0.8986±0.0204
RCNN(SimSiam-MuTF)	0.9373±0.0096	0.9193±0.0139	0.9097±0.0042	0.9083±0.0081
$\triangle$	1.67%	2.96%	0.63%	1.06%
Transformer(MSE)	0.9035±0.0067	0.8719±0.0075	0.8359±0.0143	0.8063±0.0141
Transformer(SimSiam-MuTF)	0.9321±0.0083	0.9088±0.0175	0.8633±0.0053	0.8453±0.0083
$\triangle$	3.06%	4.06%	3.17%	4.61%

Table S9 The optimal hyper-parameters in the joint training phase.

Task	Data Split Strategy		γ	δ
Binding Affinity Prediction	5-CV		0.5	0.9
	Regime		0.1	0.7
	Cold Drugs		0.7	0.5
	Cold Proteins		0.9	0.7
	Cold Both		0.9	0.5
	Sequence identities	10%	0.3	0.3
		20%	0.5	0.3
		30%	0.3	0.3
		40%	0.3	0.3
		50%	0.7	0.9
ΔAffinity Prediction	5-CV		0.1	0.9
	Regime		0.3	0.5
	Cold Drugs		0.3	0.5
	Cold Proteins		0.1	0.7
	Cold Both		0.3	0.7
	Sequence identities	10%	0.1	0.9
		20%	0.1	0.5
		30%	0.5	0.5
		40%	0.5	0.3
		50%	0.5	0.7

References

[1] Öztürk, H., Özgür, A., Ozkirimli, E.: Deepdta: deep drug–target binding affinity prediction. Bioinformatics **34**(17), 821–829 (2018)

[2] Zhao, Q., Duan, G., Yang, M., Cheng, Z., Li, Y., Wang, J.: Attentiondta: Drug–target binding affinity prediction by sequence-based deep learning with attention mechanism. IEEE/ACM transactions on computational biology and bioinformatics **20**(2), 852–863 (2022)

[3] Zhao, Q., Zhao, H., Zheng, K., Wang, J.: Hyperattentiondti: improving drug–protein interaction prediction by sequence-based deep learning with attention mechanism. Bioinformatics **38**(3), 655–662 (2022)

[4] Kayikci, M., Venkatakrishnan, A., Scott-Brown, J., Ravarani, C.N., Flock, T., Babu, M.M.: Protein contacts atlas: visualization and analysis of non-covalent contacts in biomolecules. Nature structural & molecular biology **25**(2), 185 (2018)

[5] Cha, S.-S., Lee, D., Adams, J., Kurdyla, J.T., Jones, C.S., Marshall, L.A., Bolognese, B., Abdel-Meguid, S.S., Oh, B.-H.: High-resolution x-ray crystallography reveals precise binding interactions between human nonpancreatic secreted phospholipase a2 and a highly potent inhibitor (fpl67047xx). Journal of medicinal chemistry **39**(20), 3878–3881 (1996)

[6] Dar, A.C., Lopez, M.S., Shokat, K.M.: Small molecule recognition of c-src via the imatinib-binding conformation. Chemistry & biology **15**(10), 1015–1022 (2008)

[7] Salentin, S., Schreiber, S., Haupt, V.J., Adasme, M.F., Schroeder, M.: Plip: fully automated protein–ligand interaction profiler. Nucleic acids research **43**(W1), 443–447 (2015)

[8] Gilson, M.K., Liu, T., Baitaluk, M., Nicola, G., Hwang, L., Chong, J.: Bindingdb in 2015: a public database for medicinal chemistry, computational chemistry and systems pharmacology. Nucleic acids research **44**(D1), 1045–1053 (2016)

[9] Yang, J., Roy, A., Zhang, Y.: Biolip: a semi-manually curated database for biologically relevant ligand–protein interactions. Nucleic acids research **41**(D1), 1096–1103 (2012)

[10] Mysinger, M.M., Carchia, M., Irwin, J.J., Shoichet, B.K.: Directory of useful decoys, enhanced (dud-e): better ligands and decoys for better benchmarking. Journal of medicinal chemistry **55**(14), 6582–6594 (2012)

[11] Bauer, M.R., Ibrahim, T.M., Vogel, S.M., Boeckler, F.M.: Evaluation and optimization of virtual screening workflows with dekois 2.0–a public library of challenging docking benchmark sets. Journal of chemical information and modeling **53**(6), 1447–1462 (2013)

[12] Mendez, D., Gaulton, A., Bento, A.P., Chambers, J., De Veij, M., Félix, E., Magariños, M.P., Mosquera, J.F., Mutowo, P., Nowotka, M., *et al.*: Chembl: towards direct deposition of bioassay data. Nucleic acids research **47**(D1), 930–940 (2019)

[13] Irwin, J.J., Shoichet, B.K.: Zinc- a free database of commercially available compounds for virtual screening. Journal of chemical information and modeling **45**(1), 177–182 (2005)

[14] Ferrer Florensa, A., Almagro Armenteros, J.J., Nielsen, H., Aarestrup, F.M., Clausen, P.T.L.C.: Spanseq: similarity-based sequence data splitting method for improved development and assessment of deep learning projects. NAR Genomics and Bioinformatics **6**(3), 106 (2024)
